# The role of playgrounds in promoting children’s health – a scoping review

**DOI:** 10.1186/s12966-024-01618-2

**Published:** 2024-07-08

**Authors:** Jasper Schipperijn, Cathrine Damsbo Madsen, Mette Toftager, Danielle Nørager Johansen, Ida Lousen, Thea Toft Amholt, Charlotte Skau Pawlowski

**Affiliations:** 1 World Playground Research Institute, University of Southern Denmark, Campusvej 55, 5230 Odense M, Denmark; 2 Centre for Clinical Research and Prevention, Bispebjerg and Frederiksberg Hospital, Hovedvejen 5, 2000 Frederiksberg, Denmark

**Keywords:** Playgrounds, Health benefits, Physical activity, Motor skills, Mental health, Social health

## Abstract

**Background:**

Active outdoor play is important for children’s health and development, and playgrounds provide good places for play. However, the importance of playground use for health and well-being is unclear. Our scoping review aims to create an overview of all research on playground use and health benefits for children.

**Methods:**

Scopus, Web of Science, SportDiscus, and PsycInfo were searched using two search blocks, focusing on 'playground' and 'children' respectively, for publications from 2000 to November 2023. The primary inclusion criterion was examining the relationship between playground use and positive physical, mental, or social health outcomes. Only papers published in English were reviewed. For each publication, we synthesized and condensed the results, categorizing them by playground setting, reported health outcome, participant age group, study design, methodologies, publication's country, year, and ‘stage of evidence’.

**Results:**

Data from 247 studies were extracted and nearly 80% of these publications were descriptive or exploratory studies. Fifty-two were intervention studies. Adding playground markings to schoolyards led to increased physical activity. Greening schoolyards had mainly positive effects on social and mental health. In Early Childhood Education and Care, renewing play structures had a positive effect on physical activity in three publications. All Public Open Space interventions we found were different, with mixed effects on health outcomes.

**Conclusions:**

The existing evidence provides good arguments for policy makers, city planners and school-leaders to invest in adding playground markings in schoolyards as this will likely result in more physical activity. The evidence for the health benefits of investing in new play structures indicated that tailoring the playground to local needs is important as ‘one size does not fit all’ and playgrounds need to be designed as engaging and interesting places for children’s play if they are to generate health benefits. Investing in ‘greening’ playgrounds is likely to result in social and mental health benefits for children, but does not always result in more physical activity.

The research field needs more efficacy and effectiveness studies, and in particular replication and scale-up studies to demonstrate which type of playground interventions are successful.

**Protocol:**

The review protocol was registered at Open Science Framework (https://doi.org/10.17605/OSF.IO/UYN2V).

**Supplementary Information:**

The online version contains supplementary material available at 10.1186/s12966-024-01618-2.

## Background

Every child has the right to rest, relax, play and to take part in cultural and creative activities’, as stated in article 31 of the United Nations Convention on the Rights of the Child [1]. The Convention recognizes that play is not an optional extra for children, it is fundamental to their physical, social and mental development and intrinsic to their health and happiness in the present moment. Play is considered fundamental for child development as play helps children develop social, academic, and personal competences [2]. The Millennium Cohort Study in the United Kingdom demonstrates that independent outdoor play is associated with increased moderate-to-vigorous physical activity (MVPA) and reduced sedentary time [3]. With global concerns about inadequate children's physical activity levels, only 27%–33% of children meet physical activity recommendations [4], the World Health Organization recommends children to sit less and play more to grow up healthy [5]. Following the World Health Organization definition of health [5], this means that children should thrive mentally, socially as well as physically to be healthy.

Recent decades have witnessed a decline in children's outdoor play, likely due to safety concerns, both from increased car traffic on residential streets, as well as lower levels of contact between neighbours, leading to delayed initiation of independent outdoor play [6].

A large review [7] showed that parental attitude, behaviour, support and practice are main factors influencing children’s outdoor play. Furthermore, the same review emphasised that it is important to focus on increasing outdoor play time where children can be spontaneous and creative, stimulating freely chosen and self-directed play, while focusing less on adult-led activities. Playgrounds are likely perceived as safe places for children’s play, and on average children spent more time playing at playgrounds than in any other place [6]. However, play is not only happening in children’s free time. Among the 38 OECD (Organisation for Economic Co-operation and Development) countries, on average 87% of children aged 3–5 are enrolled in Early Childhood Education and Care (ECEC) [8] and therefore ECEC centres are important settings for play. For school-aged-children, schoolyards are crucial locations for active play, contributing with up to 40% of children’s daily physical activity [9].

Numerous reviews, focusing on specific playground settings, age groups, and health parameters, have explored playground-related health outcomes. Most reviews focused on physical activity in schoolyards (e.g. Clevenger and colleagues [10]) or ECEC (e.g. Martin and colleagues [11]). A recent review and meta-analysis showed that physical activity interventions in schoolyards had a positive effect on increasing accelerometer-assessed MVPA in school-aged children [12]. Reviews have also been published on playground benefits for other physical health outcomes such as motor skills (e.g. Pawlowski and colleagues [13]), weight status (e.g. Williams and colleagues [14]) or social (e.g. Moore and colleagues [15]) and mental (e.g. Vella-Brodrick and colleagues [16]) health outcomes. Finally, some reviews looked at health benefits of using playgrounds in public open spaces (e.g. Audrey and colleagues [17]).

Because children’s play behaviour is likely to have many similarities across different playground settings, lessons learnt in one setting could potentially be applicable in other settings. Furthermore, playground interventions often have the potential to influence more than one health outcome. Having a comprehensive understanding of the overall health benefits of children’s use of playgrounds in different settings can help policy makers and city planners determine if they should prioritise investing in playgrounds﻿. It can also help health authorities, general practitioners and paediatricians decide if they should recommend parents to take their children to playgrounds regularly.

The objective of this scoping review is to identify and assess the available evidence on the health benefits of children’s use of playgrounds. More specifically, we will: 1) identify all research on playground use and health benefits, 2) assess the stage of evidence of all included publications, and 3) summarise the health effects of intervention studies of playground use.

## Methods

The scoping review was conducted in accordance with the JBI methodology for scoping reviews [18]. The review protocol was registered at Open Science Framework (10.17605/OSF.IO/UYN2V) in May 2022 and the PRISMA guidelines for scoping reviews were followed in designing, conducting, and reporting the results. The search was updated in November 2023.

### Information sources and search strategy

In May 2022, we initiated a systematic search in four electronic databases: Scopus, Web of Science, SportDiscus, and PsycInfo. Collaborating with a research librarian, authors tested and refined search terms. To ensure comprehensive coverage, a sensitive search strategy was devised with two blocks—one containing synonyms for 'playground' and the other for 'children'. The search terms for Scopus are outlined in Table 1. Slight adaptations were made for each database's specific requirements. The updated search looked at the same four databases to include articles from 2022 and 2023. Prior to full-text screening of new articles, 2022 publications assessed in the original search were excluded as "Already included or excluded".

**Table 1:** Search terms for Scopus

(TITLE (playground*)) OR (((TITLE-ABS (schoolyard* OR "school ground*") OR AUTHKEY (schoolyard* OR "school ground*")) OR (TITLE-ABS (play W/3 (area* OR space* OR environment* OR field* OR natural OR nature OR outdoor OR place* OR structure* OR equipment OR park* ) ) OR AUTHKEY ( play W/3 ( area* OR space* OR environment* OR field* OR natural OR nature OR outdoor OR place* OR structure* OR equipment OR park* ) ) ) OR ( TITLE-ABS ( ( school* OR daycare* OR "day care" OR childcare OR "child care" OR kindergarten* ) W/6 ( play OR playable OR played OR playing OR "physical* activ*" OR "organi?ed activit*" OR "unorgani? ed activit*" OR "structured activit*" OR "unstructured activit*" OR "recreation* activit*" OR "leisure activit*" OR "outdoor activit*" OR "vigorous activit*" ) ) OR AUTHKEY ( ( school* OR daycare* OR "day care" OR childcare OR "child care" OR kindergarten* ) W/6 ( play OR playable OR played OR playing OR "physical* activ*" OR "organi? ed activit*" OR "unorgani? ed activit*" OR "structured activit*" OR "unstructured activit*" OR "recreation* activit*" OR "leisure activit*" OR "outdoor activit*" OR "vigorous activit*" ) ) ) OR ( TITLE-ABS ( playfield* OR playplace* OR playscape* OR playspace* OR "public open space" ) OR AUTHKEY ( playfield* OR playplace* OR playscape* OR playspace* OR "public open space" ) ) OR ( INDEXTERMS ( playground ) ) OR ( ABS ( playground* ) OR AUTHKEY ( playground* ) ) ) AND ( ( TITLE-ABS ( adolescen* OR baby OR boy OR schoolboy* OR boyhood OR girlhood OR child* OR schoolchild* OR girl OR schoolgirl* OR infan* OR juvenil* OR kid OR minor OR newborn* OR new-born* OR paediatric* OR pediatric* OR preschool* OR puber* OR pubescen* OR teen* OR tween* OR toddler* OR youth* OR student* OR schoolage* ) OR AUTHKEY ( adolescen* OR baby OR boy OR schoolboy* OR boyhood OR girlhood OR child* OR schoolchild* OR girl OR schoolgirl* OR infan* OR juvenil* OR kid OR minor OR newborn* OR new-born* OR paediatric* OR pediatric* OR preschool* OR puber* OR pubescen* OR teen* OR tween* OR toddler* OR youth* OR student* OR schoolage* ) ) OR ( INDEXTERMS ( child ) OR INDEXTERMS ( adolescent ) OR INDEXTERMS ( pediatric ) ) ) )	

### Eligibility criteria

Playgrounds were defined as *places designed or designated to facilitate play*. We included both indoor and outdoor playgrounds, public playgrounds, school playgrounds, ECEC playgrounds, as well as private playgrounds, and playgrounds requiring payment. Publications that exclusively focused on unfixed equipment or sports facilities, were excluded, as were studies exclusively focusing on organising play activities, and studies on temporary playstreets.

The primary inclusion criterion was examining the relationship between playground usage and positive physical, mental, or social health outcomes. Studies focusing on negative health outcomes (like injuries and bullying), environmental exposures (such as pollution, pesticides, sun exposure), playground availability, quality, safety, or security were excluded.

All children and adolescent populations (aged 0–17 years) were considered, irrespective of gender, health status, or physical abilities. Peer-reviewed articles published in English from January 2000 to November 2023 were included, while guidelines, conference abstracts, protocols, book chapters, PhD dissertations, reviews, and methodological papers were excluded. The year 2000 was chosen as a start year to balance being comprehensive with the relevance of publications to inform future research. An initial pilot screening in June 2022 ensured a consistent understanding and application of the inclusion criteria among authors.

### Selection process

All publications were imported into Endnote 20.0.1, and duplicates were removed, before transferring all data to Covidence for screening of titles and abstracts. The full texts of the included publications were independently assessed by two authors between August and October 2022. Any discrepancies arising from decisions about inclusion or exclusion of a publication were addressed through discussions moderated by authors JS or CSP. The process was repeated in November 2023 for the updated search.

To assess the sensitivity of our search strategy, one author (CDM) examined if we had overlooked potentially relevant publications by screening the reference lists of ten randomly selected included full-text publications for any potentially relevant additional publications. This process took place in January 2023 and did not result in additional publications being identified.

### Data extraction process and data items

Data were extracted from all included publications by two student assistants and one of the authors (CDM), and cross-checked by JS, CSP, and MT to ensure accuracy and consistency. We extracted data on authorship, country of origin, World Bank country income level [19], year of publication, research aim, number of participants involved, setting, health outcomes examined, study design, methodologies employed, and key findings.

### Synthesis of extracted results

We encountered multiple publications from the same study and therefore our reporting is structured per publication rather than per study. For each publication, we synthesized and condensed the results, categorizing them by playground setting, reported health outcome, participant age group, study design, methodologies, publication's country, year, and ‘stage of evidence’.

Health outcomes were categorised as: physical health (subdivided in physical activity, motor skills, and weight status), social health (e.g. interactions with peers, social network), or mental health (e.g. well-being, self-esteem, and cognitive health outcomes).

The playground settings were categorised into four main contexts: 1) ECEC (i.e. day-care, kindergarten, and pre-school), 2) School (i.e. primary, elementary, middle, and/or secondary school), 3) Public open spaces (e.g. parks, squares with public playgrounds), and 4) Healthcare (e.g. hospitals, rehabilitation centre, or facilities for children with special needs).

The broad target population of children and adolescents (aged 0–17) was divided into the following age groups: 0-2yrs (toddlers), 3-5yrs (early childhood), 6-12yrs (middle childhood), and 13-17yrs (adolescents).

Following Bauman and Nutbeam [20], we assume that there are multiple stages of evidence in relation to the evaluation of health promotion programs. Various types of *descriptive and exploratory studies* are primarily used to define the problem (stage 1), before developing possible solutions that are tested in *feasibility and pilot studies* (stage 2), followed by *efficacy and effectiveness studies* (stage 3), and *replication studies* studying the implementation and effectiveness of interventions in a different context (stage 4), before *scale-up studies* (stage 5), and eventually *monitoring studies* of fully implemented health promotion programs can take place (stage 6). For each included publication the ‘stage of evidence’ was assigned independently by authors JS and CSP, and initial inconstancies were discussed and resolved.

For publications reporting results from intervention studies, we described and categorised the main intervention components, and summarised the findings by classifying the effect of playground use on health outcomes as: positive, negative, or showed no effect. If a publication reported on the same outcome measured with multiple methods or for multiple sub-groups, with a conflicting direction of the effect, the outcome was labelled as 'inconclusive'.

## Results

### Publication selection

**Fig. 1:**
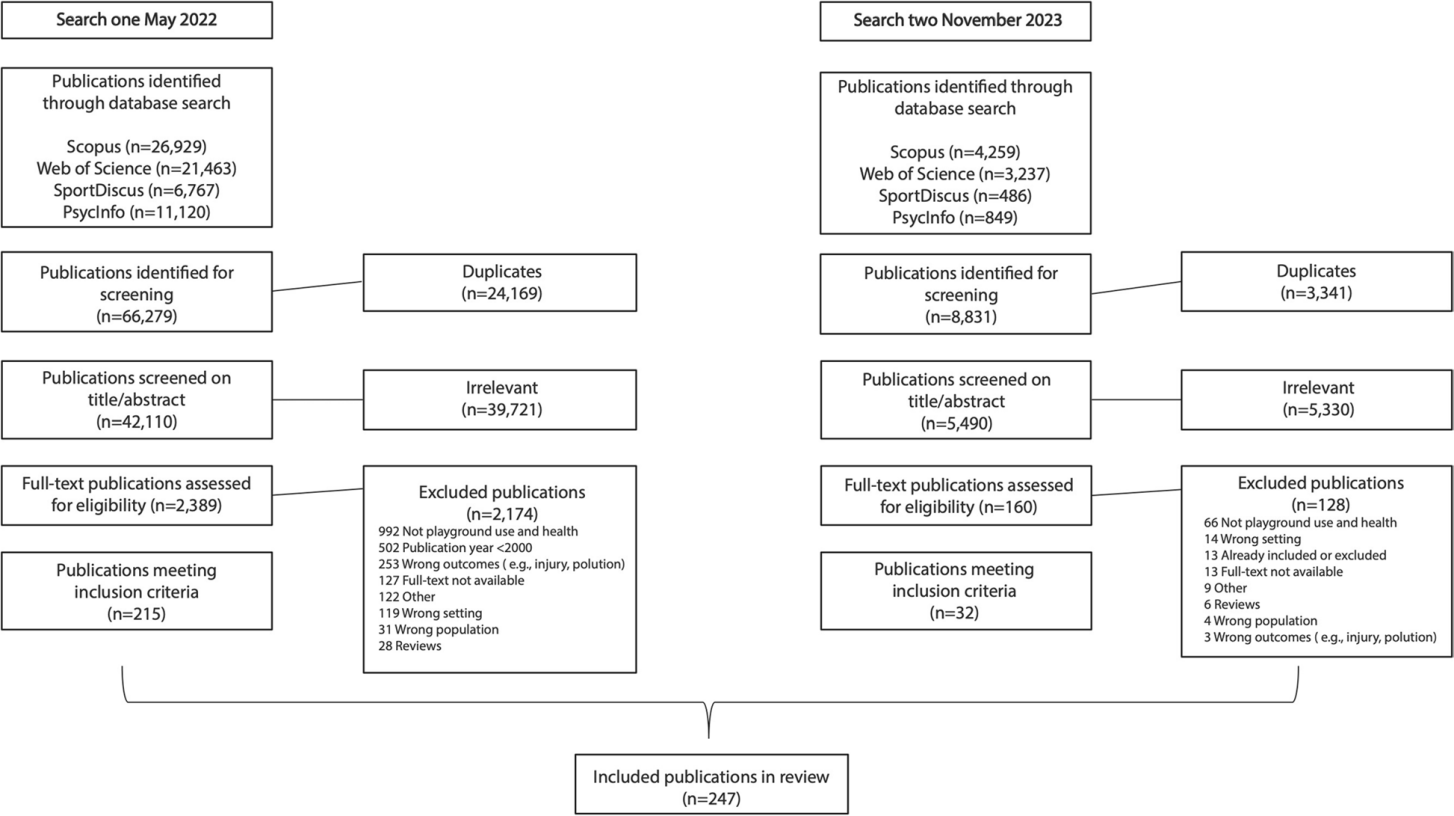
PRISMA flowchart

The initial search across the four databases yielded a total of 66,279 potentially relevant publications. After removing duplicates, the titles, and abstracts of 42,110 publications were screened, and 2,389 publications were selected for full-text screening. Following the full-text screening process, 215 publications met the inclusion criteria and were included in this scoping review. The search update resulted in 8,831 potentially relevant publications. After duplicates were removed 5,490 publications remained for title/abstract screening of which 5,330 were excluded, leaving 160 for full-text screening, resulting in 32 additional publications that met the inclusion criteria. In total, data extracted from 247 publications formed the basis of this scoping review. A full reference list of all 247 publications can be found in Additional file 1, and data extracted from all 247 publications can be found in Additional file 2. See Fig. 1 for the PRISMA flowchart of our selection process.

### Publication characteristics

Three-out-of-four (75.7%) of the 247 included publications were published after 2012. Almost all publications reported on studies in high-income countries (92.7%), 117 were from Europe (47.4%), 81 were from North America (32.8%), and 33 were from Oceania (13.4%). Eighteen publications were from low- and middle-income countries (LMIC), whereof three were from Africa, five from Central and South America, and eight from Asia. Out of the 247 publications, 22 included 0–2-year-old children (8.9%), 107 included 3–5-year-old children (43.3%), 169 included 6–12-year-old children (68.4%), and 42 included 13–17-year-old children (17.0%). Only 11 of the 247 publications (also) included children and adolescents living with disability.

Most publications (*n* = 195, 78.9%) reported results from descriptive or exploratory studies, 31 (12.6%) presented results from feasibility or pilot studies, 20 (8.1%) were based on efficacy and effectiveness studies, and one was based on a replication study. More than half of the publications (*n* = 130, 52.6%) reported on studies that took place in a schoolyard playground, 63 were in an ECEC playground (25.5%), 54 (21.9%) in a public open space, and three studies took place on a playground in connection with a healthcare centre (1.2%). See characteristics of included publications in Table 2.

**Table 2:** Characteristics of included publications. *N* = 247

	n	%
**Geography**^a^		
Europe	117	47.4%
North America	81	32.8%
Oceania	33	13.4%
Asia	13	5.3%
South America	4	1.6%
Africa	3	1.2%
**Income level**		
High	229	92.7%
Low- and middle-income countries	18	7.3%
**Playground Setting**^a^		
ECEC	63	25.5%
School	130	52.6%
Public Open Space	54	21.9%
Healthcare	3	1.2%
**Age group**^a^		
0-2yrs (toddlers)	22	8.9%
3-5yrs (early childhood)	107	43.3%
6-12yrs (middle childhood)	169	68.4%
13-17yrs (adolescents)	42	17.0%
**Population**^a^		
Living with disabilities	11	4.5%
General population	237	96.0%
**Outcomes reported**^a^		
Physical activity	192	77.7%
Motor skills	15	6.1%
Weight status	12	4.9%
Social health	54	21.9%
Mental health	30	12.1%
**Methods used**^a^		
Observation	114	46.2%
Wearable device	96	38.9%
Survey	60	24.3%
Interview	58	23.5%
Physical assessment	19	7.7%
Camera	38	15.4%
Other	9	3.6%
**Stage of evidence**		
Descriptive and exploratory studies (stage 1)	195	78.9%
Feasibility and pilot studies (stage 2)	31	12.6%
Efficacy and effectiveness studies (stage 3)	20	8.1%
Repletion studies (stage 4)	1	0.4%
Scale-up studies (stage 5)	0	0
Monitoring studies (stage 6)	0	0

^a^One publication can include multiple geographical locations, settings, population and age groups, outcomes, and methods. For that reason, the numbers in each of these categories do not sum to the overall number

### Results reported in the included publications

Physical activity was the primary outcome in 192 publications, followed by social health (*n* = 54), mental health (*n* = 30), motor skills (*n* = 15), and weight status (*n* = 12), see Table 3. A full overview of the results reported in all included publications can be found in Additional file 2.

**Table 3:** Number of publications reporting on each health outcome, grouped by settings and by evidence stage. *N* = 303 health outcomes in 247 publications

	*Physical health*			*Social health*	*Mental health*	Number of publications*
	Physical activity	Motor skills	Weight status			
**Number of publications**	**192**	**15**	**12**	**54**	**30**	**247**
*School*						
Descriptive and exploratory studies	78	1	5	22	6	**98**
Feasibility and pilot studies	12	0	0	4	3	**14**
Efficacy and effectiveness studies	16	0	2	3	3	**17**
Repetition studies	1	0	0	0	0	**1**
**School overall**^a^	**107**	**1**	**7**	**29**	**12**	**130**
*ECEC*						
Descriptive and exploratory studies	35	6	2	12	10	**51**
Feasibility and pilot studies	7	2	0	1	2	**9**
Efficacy and effectiveness studies	2	0	0	0	0	**2**
Repetition studies	0	0	0	0	0	0
**ECEC overall**^a^	**44**	**8**	**2**	**13**	**12**	**62**
*Public Open Space*						
Descriptive and exploratory studies	37	2	3	10	6	**46**
Feasibility and pilot studies	5	2	0	1	0	**8**
Efficacy and effectiveness studies	1	0	0	0	0	**1**
Repetition studies	0	0	0	0	0	0
**Public open space overall**^a^	**43**	**4**	**3**	**11**	**6**	**55**
*Healthcare*						
Descriptive and exploratory studies	0	2	0	2	1	**3**
Feasibility and pilot studies	0	0	0	0	0	0
Efficacy and effectiveness studies	0	0	0	0	0	0
Repetition studies	0	0	0	0	0	0
**Healthcare overall**^a^	0	**2**	0	**2**	**1**	**3**

*ECEC *Early Childhood Education and Care

^a^One publication can include multiple outcomes, and multiple settings. For that reason, the numbers in each row, for each outcome, for each stage of evidence, do not necessarily sum to the overall numbers

### Effects of using playgrounds – results from intervention studies

Presenting and summarizing all results from 247 publications in one review paper is not feasible, so we have chosen to focus on the stronger (stage 2 and higher) evidence presented in 52 intervention studies. Five focused on the health effects of free outdoor play, while 24 explored the impact of new playground structures—sometimes with additional elements like markings, loose equipment (e.g. balls, rackets, bats, skipping ropes), activities (e.g. sports or games), or staff training (e.g. on how to encourage children to be physically active during recess). The added play structures varied widely in type and budget. Playground markings were studied in 16 publications, and eight examined the effect of (access to) nature, often through greening school playgrounds. Additionally, six publications explored unique interventions, such as removing benches, lowering schoolyard density, opening the schoolyard after school hours, or comparing sports fields with playgrounds.

### School

Most intervention studies were conducted in the school setting (one repetition, 17 efficacy and effectiveness, and 14 feasibility and pilot studies). The primary intervention components studied were play structures (16 publications) and playground markings (14 publications), often combined with each other, or integrated with organised activities or loose equipment.

#### Play structures in the schoolyard

Numerous school-based studies investigated the impact of renovating schoolyards with new play structures, greatly varying in type and size. The Danish SPACE study [21] and 'Activating Schoolyards' project [22] both included several new innovative play structures and landscaping developed after consulting school children, but results varied. Positive associations between the perceived schoolyard and physical activity were found in the SPACE study [21] while the Activating Schoolyards study reported increased activity for the least active children [22] but mixed effects in a sub-sample of all children [23]. In a Swedish study, adding play structures and landscaping during the renovations primarily attracted already active children, with less active ones being spectators [24]. The 'Camden active spaces' project in London showed insignificant effects on physical activity levels after renovating schoolyards in a deprived area where each school received a unique playground design, e.g., including new AstroTurf games pitches, climbing frames, trampolines, monkey bars, and outdoor gyms, which were designed based on themes emerging from consultations with children [25]. A study with a small (2000 Euro) intervention budget in the Netherlands [26] and a large natural experiment in Cleveland, USA [27], did not demonstrate significant effects on accelerometer-measured physical activity, but the Cleveland study did show a significant increase in schoolyard use. Conversely, a study in Denver, USA, showed increased physical activity levels after renovating schoolyards as part of the ‘Learning Landscapes’ program. Each schoolyard had unique attributes, but also common elements including areas with age-appropriate play equipment, asphalt areas for structured games such as basketball and tetherball and a grassed multipurpose playfield, typically with a track. All of the schoolyards had a central gathering space with a shade structure. Trees were planted in hard surface and grassed areas to increase shade [28]. In a small study at a school in Leadville, Colorado showed a positive effect after a renewal process added six swings, a mesh climbing structure, slides, and a spinning carousel. A new outdoor basketball court, walkways, boulder retaining walls, and grass-covered open play area were also constructed. Additional loose equipment was provided during post-observations, including balls, hula hoops, and cones for creating a course [29].

In a study focusing on eight boys with autism spectrum disorder, moving to a new playground designed for enhanced social interactions significantly increased group play and social interactions [30].

### Playground markings in schoolyards

Feasibility and pilot studies examining the impact of adding playground markings in schoolyards were conducted in the UK [31, 32] the USA [33] and Spain [34] all demonstrating positive effects on physical activity. Typically, the markings consisted of a combination of game-related marking (e.g. hopscotch or 4-square), fantasy element (e.g. castles, dragons, snakes or animals), educational markings (e.g. clock faces or letter squares), or mazes and trails. In an efficacy and effectiveness study in the Northwest of England, Ridgers and colleagues reported a positive effect on recess physical activity after 6 weeks, a significant effect after 6 months, and no significant effect after 12 months [35, 36]. A study in Mexico City [37] found a positive effect on physical activity with basic and comprehensive interventions involving markings, loose equipment, and organised activities. The Australian Transform-Us! intervention showed a significant mid-intervention effect on recess physical activity [38]. A French study by Baquet et al. [39] reported a positive effect of playground markings on physical activity after 6 and 12 months. Crust and colleagues [40] found no influence on children’s physical self-perception but observed positive effects on physical activity and pro-social behaviour with added markings. Benthroldo et al. [41] in Brazil did not find a significant effect on self-reported physical activity after adding markings and loose equipment to public schools. Finally, a repetition study in France [42] reported a significant positive effect on accelerometer-measured physical activity using the same markings intervention as Ridgers and colleagues [35, 36] in the UK.

#### Natural elements (greening) in the schoolyard

Adding more natural elements (greening) in schoolyards was investigated in five feasibility and pilot studies. Barton et al. [43] compared loose equipment provision with a nature-based orienteering intervention, showing greater physical activity increase in the schoolyard than the orienteering area. Amicone et al. [44] found improved attention and perceived restorativeness in a green schoolyard compared to a paved surface schoolyard. Wood et al. [45] compared physical activity levels in the schoolyard versus the school field, finding children were less active (accelerometer measured) in the schoolyard than the school sports field.

A Los Angeles County pilot study [46] reported increased physical activity after replacing asphalt with green space at three time points. Similar schoolyard renovations in Chicago [47] led to heightened physical activity and prosocial interactions. A Dutch study [48] replacing pavement with greenery on schoolyards showed positive effects on attentional restoration, social well-being, and increased accelerometer-measured physical activity for girls.

### Early Childhood Education and Care (ECEC)

In ECEC settings, nine publications reported on feasibility and pilot intervention studies, while two reported on efficacy and effectiveness studies. Three studies examined the feasibility of free outdoor play. MacArthur et al. [49] compared 15-min of unstructured outdoor play with 15-min of active video games using an Xbox 360 Kinect, showing inconclusive results on accelerometer measured physical activity. Tandon et al. [50] found no significant impact when comparing free outdoor play to teacher-led play. Lundy and Trawick-Smith [51] found a positive association between naturalistic playground play (i.e. free play, as opposed to adult-directed play) and on-task behaviour for boys, as well as children of low socio-economic status.

Canadian research [52] on increasing nature and risky play opportunities (play classified as: rough and tumble, height, mastery, unstable, speed, risk of getting lost) in ECEC settings showed significant decreases in accelerometer-measured physical activity and inconclusive results for social behaviours. A San Diego study observed increased activity levels after renovating a university ECEC outdoor playground, but no change in accelerometer-measured activity levels [53]. A large Belgian study [54] showed no increase in accelerometer-measured recess physical activity with added markings or loose equipment. Conversely, a Belgian pilot study [55] that varied recess times so that the number of children in the playground during recess was smaller, showed increased accelerometer-measured physical activity. A Japanese pilot study [56] reported a significant increase in accelerometer-measured physical activity by changing the layout of an ECEC playground to separate playground elements more, and make sure that play in one area did not disturb play in another area. Lastly, a natural experiment in Perth, Australia [57] showed significant increases in accelerometer-measured physical activity after renovating six ECEC outdoor playgrounds compared to unchanged ones.

Webster et al. [58] found no effect of adding playground markings and staff training on fundamental motor skills or physical activity in a US pilot study. And a small Norwegian study [59] on nature's impact on motor skills yielded mixed results.

### Public open space

Our search yielded eight feasibility and pilot studies on playgrounds in public open spaces and one publication reporting on efficacy and effectiveness. The REVAMP natural experiment in Melbourne, Australia, found a positive impact on observed physical activity with the construction of a large playscape with many different play structures and landscaping [60]. A study in Sydney, Australia, showed increased physical activity for boys, but not girls, in a renovated park playground with added play structures [61]. Farley et al. [62] observed enhanced neighbourhood physical activity after opening a schoolyard for use after school hours in a low-income neighbourhood in New Orleans, USA. Roemmich et al. [63] found increased physical activity for both children and parents after removing park benches around a playground in another US study. A Danish study by Pawlowski et al. [64] evaluated the effect of co-creating a new neighbourhood playground with local 10–11-year-olds but did not observe an increase in playground use and activity levels. Molenberg et al. [65] evaluated adding 13 new activity and play spaces to low-income neighbourhoods in the Netherlands but did not show a significant effect on physical activity.

Tortella et al. [66] reported significant improvement in four out of six gross motor skills in a study of a new playground in Northern Italy designed to enhance fundamental motor skills. However, in a subsequent study [67], comparing free play and partly structured activity at the new playground showed no difference in motor skills between the two groups. Yang et al. [68] observed increased peer interactions after adding a play structure in a large park in Taipei, Taiwan.

## Discussion

We set out to review and synthesise evidence on the health benefits of children's playground use. After assessing over 47,000 titles and abstracts, we extracted data from 247 included publications. Nearly 80% of these publications were descriptive or exploratory studies (evidence stage 1). Fifty-two were intervention studies, with 31 reporting on feasibility or pilot studies (stage 2), 20 reporting on efficacy and effectiveness studies (stage 3), and one reporting on a replication study (stage 4). Physical activity was the predominant health outcome studied, followed by social and mental health. Most intervention studies were conducted at schools, followed by ECEC, and Public Open Spaces. Three studies, all descriptive or exploratory (stage 1), were conducted in healthcare settings. Over 90% of all publications included were conducted in high-income countries, which limits the generalisability of currently available evidence.

The longitudinal analysis of data from the Gateshead Millennium Cohort Study revealed that the total volume of physical activity already starts declining by age 7 in the UK for both boys and girls, and, unlike many other studies, that this decline did not intensify during adolescence [69]. This emphasises the importance of implementing physical activity promotion interventions during primary-school age. One successful intervention, tested in various countries, is the addition of playground markings [31–42]. Renovating schoolyards with new play structures, though varied in budget, and yielding mixed results, also showed several positive effects [21, 22, 24, 28–30]. Greening schoolyards had positive effects on physical activity [46–48], as well as social and mental health [44, 47, 48].

In ECEC, providing more space per child had a positive impact on physical activity in a pilot study [55]. However, greening an ECEC playground had a negative effect on physical activity but a positive effect on social health [52]. In contrast to schools, adding playground markings in ECEC did not increase physical activity [58]. Renewing play structures in ECEC had a positive effect on physical activity in three publications [55–57].

All Public Open Space interventions we found were different, and even though many added play structures, they were not directly comparable, and results were mixed. Opening schoolyards for use by neighbourhood children outside of school hours increased activity levels [62], as did the construction of a large playscape in a park [60].

Our scoping review underscores the evidence supporting health benefits from playground use, but the effectiveness of interventions varies by setting, health outcome, and intervention component. Schoolyard markings, a cost-effective intervention, exhibit predominantly positive effects on children’s physical activity, warranting a logical next step—a scale-up study (evidence stage 5) like the one planned for the Australian 'Transform-Us!' program [70]. However, evidence for mental and social health outcomes is less abundant, highlighting the need for more comprehensive schoolyard studies. The diverse interventions adding play structures to schoolyards resulted in unclear health effects, necessitating studies that describe the interventions, and the program theory behind them, in (much) more detail, and include robust implementation measures so that the mechanisms can be better understood.

Motor skills in preschool-aged children from high-income countries are insufficient [71], impacting physical activity and weight status negatively across the lifespan [72]. Only three feasibility and pilot studies (stage 2) have explored how ECEC playgrounds influence motor skills. Larger scale (stage 3) studies are needed to provide evidence towards recommendations for motor skill development in ECEC. The planned scale-up of the Play Active intervention [73] in Australia will hopefully be able to provide stage 5 evidence of the health effects of a multi-component ECEC intervention.

The evidence for health benefits related to playgrounds in public open space playgrounds is less convincing with 47 out of the 55 included publications being based on descriptive and exploratory (stage 1) studies. Stage 2 and 3 intervention studies with a robust design, encompassing multiple health outcomes, are needed before evidence-based recommendations can be established.

Based on the playground descriptions and illustrations included in the publications we assessed, there is a very large variation in playground design Various publications mentioned that interventions need to be tailored to local needs and possibilities, ‘one size does not fit all’ when designing playground interventions, and evaluation studies need to take this tailoring process into account. A good playground renovation most likely needs to start with a thorough assessment of the current situation, to make sure that the additional play structures add variation and provide new opportunities for children that were not already catered for. Most studies mentioned consulting children before redesigning, and some studies actively involved children in the co-design of places to play. In general, involvement and co-design was mentioned as something positive but a Danish study [64] that specifically focused on evaluating the co-design process showed a negative effect on the use and activity after renewal. Furthermore, the fact that playgrounds can be designed in many ways, needs to be studied in more detail. For example, a pilot study found that the spatial layout of playground affects the pattern of play activity and the physical activity levels of young children [56].

### Strengths and limitations

A scoping review is useful to map the literature on evolving or emerging topics and to identify gaps [74]. We followed the JBI methodology and PRISMA guidelines for scoping reviews for a robust, rigorous, and transparent review protocol [75], thus the risk of bias in our review methodology is low. However, despite an extensive assessment alignment process, reviewing 47,600 titles and abstracts as a team may have caused some inconsistencies in the selection process. A strength is that the search procedure was developed by a research team of experts in the research field of playground usage in collaboration with a librarian with extensive expertise in search strategies for scoping reviews. To capture as much relevant research as possible, four different databases were searched. However, given the large number of publications retrieved, we questioned if we should have created a third block containing health outcomes to narrow-down our search, but a search that is too narrow may compromise the breadth and depth of the review and is not suggested in the literature around scoping reviews [74]. Also, in accordance with doing scoping reviews, no strict quality assessment of included publications was performed since we wanted to cover all knowledge on the subject regardless of the design and quality of the study to create an overview of the research field. We did, however, assess the ‘stage of evidence’ for all publications, which, in our opinion, gives a good indication of how strong the evidence presented is. We did not include studies’only’ focusing on playground use or factors influencing playground use without measuring a health outcome. In future, these studies are also important to review to understand the mechanisms behind increasing playground use. Furthermore, while we used an inclusive definition of health, we did not include studies with relevant non-health outcomes, e.g. learning outcomes.

Finally, we only included positive physical, mental, or social health outcomes, assuming that almost all new or renovated playgrounds are safe, while challenging and fun for children. For a full overview of all health effects of using playgrounds, also negative health outcomes (like injuries and bullying), and environmental exposures (such as pollution, pesticides, sun exposure), should be assessed.

## Conclusions

This scoping review builds on data extracted from 247 publications, demonstrating that there is a lot of research on the health benefits of playgrounds. However, most publications (nearly 80%) were based on descriptive or exploratory studies. We did include 52 intervention studies, but the majority were feasibility or pilot intervention studies, indicating that the research field needs more efficacy and effectiveness studies, and in particular replication and scale-up studies. However, the existing evidence already provides good arguments for policy makers, city planners and school-leaders to invest in adding playground markings in schoolyards as this will likely result in more physical activity. The evidence for the health benefits of investing in new play structures indicated that tailoring the playground to local needs is important as ‘one size does not fit all’ and playgrounds need to be designed as engaging and interesting places for children’s play if they are to generate health benefits. Investing in ‘greening’ playgrounds is likely to result in social and mental health benefits for children.

Providing available playgrounds are safe, health authorities, general practitioners and paediatricians can recommend parents to take their children to playgrounds regularly as using playgrounds will increase physical activity levels and stimulate social interaction with other children.

### Supplementary Information

Supplementary Material 1.

Supplementary Material 2.

Supplementary Material 3.

## Data Availability

All data extracted from the 247 included publications can be found in Additional file 2. Furthermore, all publications included in this scoping review can be found in a searchable database on the website of the World Playground Research Institute. See https://playgroundresearch.org/article-database/ For selected publications and topics, short summaries were created as ‘briefs’, which are also available on the website, see https://playgroundresearch.org/research-briefs/

## Abbreviations

ECEC: Early Childhood Education and Care
MVPA: Moderate-to-Vigorous Physical Activity
