# Supplementary material for: The role of playgrounds in promoting children’s health – a scoping review
Source: Int J Behav Nutr Phys Act. 2024 Jul 8;21:72. doi: 10.1186/s12966-024-01618-2 (PMC11232259; doi:10.1186/s12966-024-01618-2)
Supplement: Supplementary file 1 — Supplementary Material 1. [file 12966_2024_1618_MOESM1_ESM.docx]

Additional file 1 - Full referencelist of all included papers

1. Aburto NJ, Fulton JE, Safdie M, Duque T, Bonvecchio A, Rivera JA. Effect of a school-based intervention on physical activity: Cluster-randomized trial. *Med Sci Sports Exerc* 2011;**43**(10):1898-906.<https://doi.org/10.1249/MSS.0b013e318217ebec>

2. Adams J, Veitch J, Barnett L. Physical activity and fundamental motor skill performance of 5–10 year old children in three different playgrounds. *Int J Environ Res Public Health* 2018;**15**(9).<https://doi.org/10.3390/ijerph15091896>

3. Akpinar A. Green Exercise: How Are Characteristics of Urban Green Spaces Associated with Adolescents' Physical Activity and Health? *Int J Environ Res Public Health* 2019;**16**(21).<https://doi.org/10.3390/ijerph16214281>

4. Alcantara-Porcuna V, Sanchez-Lopez M, Martinez-Andres M, Martinez-Vizcaino V, Ruiz-Hermosa A, Rodriguez-Martin B. Teachers' perceptions of barriers and facilitators of the school environment for physical activity in schoolchildren: a qualitative study. *Qualitative Research in Sport Exercise and Health* 2022.<https://doi.org/10.1080/2159676x.2022.2037696>

5. Alcántara-Porcuna V, Sánchez-López M, Martínez-Vizcaíno V, Martínez-Andrés M, Ruiz-Hermosa A, Rodríguez-Martín B. Parents’ perceptions on barriers and facilitators of physical activity among schoolchildren: A qualitative study. *Int J Environ Res Public Health* 2021;**18**(6):1-27.<https://doi.org/10.3390/ijerph18063086>

6. Alme H, Reime MA. Nature kindergartens: a space for children’s participation. *Journal of Outdoor and Environmental Education* 2021;**24**(2):113-31.<https://doi.org/10.1007/s42322-021-00081-y>

7. Amholt TT, Dalgas BW, Veitch J, et al. Motivating playgrounds: Understanding how school playgrounds support autonomy, competence, and relatedness of tweens. *International Journal of Qualitative Studies on Health and Well-being* 2022;**17**(1).<https://doi.org/10.1080/17482631.2022.2096085>

8. Amholt TT, Jespersen JF, Zacho M, Timperio A, Schipperijn J. Where are tweens active in school playgrounds? A hot-spot analysis using GPS, accelerometer, and GIS data. *Landscape and Urban Planning* 2022;**227**.<https://doi.org/10.1016/j.landurbplan.2022.104546>

9. Amholt TT, Pawlowski CS, Jespersen JF, Schipperijn J. Investigating the use of playgrounds by tweens: A systematic observation study. *International Journal of Play* 2022;**11**(4):363-81.<https://doi.org/10.1080/21594937.2022.2136468>

10. Amicone G, Petruccelli I, De Dominicis S, et al. Green Breaks: The restorative effect of the school environment's green areas on children's cognitive performance. *Front Psychol* 2018;**9**(OCT).<https://doi.org/10.3389/fpsyg.2018.01579>

11. Aminpour F. From the child's perspective: How the layout of outdoor school environments shapes conflict between children's self-directed play1. *J Environ Psychol* 2022;**79**.<https://doi.org/10.1016/j.jenvp.2021.101727>

12. Aminpour F, Bishop K. Children's preferences on the move: Establishing the characteristics of unofficial paths and their benefits for children's physical play in Australian primary school grounds. *J Environ Psychol* 2021;**75**.<https://doi.org/10.1016/j.jenvp.2021.101599>

13. Aminpour F, Bishop K, Corkery L. The hidden value of in-between spaces for children's self-directed play within outdoor school environments. *Landscape and Urban Planning* 2020;**194**.<https://doi.org/10.1016/j.landurbplan.2019.103683>

14. Amo C, Prochnow T. An iSOPARC Case Study of Physical Activity at a Pop-Up Park. *Res Q Exerc Sport* 2023.<https://doi.org/10.1080/02701367.2023.2265443>

15. Andersen HB, Christiansen LB, Pawlowski CS, Schipperijn J. What we build makes a difference – Mapping activating schoolyard features after renewal using GIS, GPS and accelerometers. *Landscape and Urban Planning* 2019;**191**.<https://doi.org/10.1016/j.landurbplan.2019.103617>

16. Andersen HB, Klinker CD, Toftager M, Pawlowski CS, Schipperijn J. Objectively measured differences in physical activity in five types of schoolyard area. *Landscape and Urban Planning* 2015;**134**:83-92.<https://doi.org/10.1016/j.landurbplan.2014.10.005>

17. Anthamatten P, Brink L, Lampe S, Greenwood E, Kingston B, Nigg C. An assessment of schoolyard renovation strategies to encourage children's physical activity. *Int J Behav Nutr Phys Act* 2011;**8**.<https://doi.org/10.1186/1479-5868-8-27>

18. Anthamatten P, Fiene E, Kutchman E, et al. A microgeographic analysis of physical activity behavior within elementary school grounds. *Am J Health Promot* 2014;**28**(6):403-12.<https://doi.org/10.4278/ajhp.121116-QUAN-566>

19. Baek S, Raja S, Park J, Epstein LH, Yin L, Roemmich JN. Park design and children’s active play: a microscale spatial analysis of intensity of play in Olmsted's Delaware Park. *Environment and Planning B: Planning and Design* 2015;**42**(6):1079-97.<https://doi.org/10.1177/0265813515599515>

20. Bagot KL, Allen FCL, Toukhsati S. Perceived restorativeness of children's school playground environments: Nature, playground features and play period experiences. *J Environ Psychol* 2015;**41**:1-9.<https://doi.org/10.1016/j.jenvp.2014.11.005>

21. Bai P, Schipperijn J, Rosenberg M, Christian H. Where are preschoolers active in childcare centers? A hot-spot analysis using GIS, GPS and accelerometry data. *Children's Geographies* 2023;**21**(4):660-76.<https://doi.org/10.1080/14733285.2022.2104627>

22. Bai PL, Schipperijn J, Rosenberg M, Christian H. Neighborhood Places for Preschool Children's Physical Activity A Mixed-Methods Study Using Global Positioning System Geographic Information Systems, and Accelerometry Data. *Journal of Physical Activity & Health* 2023.<https://doi.org/10.1123/jpah.2022-0462>

23. Baines E, Blatchford P. Sex differences in the structure and stability of children's playground social networks and their overlap with friendship relations. *Br J Dev Psychol* 2009;**27**(3):743-60.<https://doi.org/10.1348/026151008X371114>

24. Bao Y, Gao M, Luo D, Zhou X. The influence of outdoor play spaces in urban parks on children's social anxiety. *Frontiers in Public Health* 2022;**10**.<https://doi.org/10.3389/fpubh.2022.1046399>

25. Bao Y, Gao M, Luo D, Zhou XD. Urban Parks-A Catalyst for Activities! The Effect of the Perceived Characteristics of the Urban Park Environment on Children's Physical Activity Levels. *Forests* 2023;**14**(2).<https://doi.org/10.3390/f14020423>

26. Baquet G, Aucouturier J, Gamelin FX, Berthoin S. Longitudinal Follow-Up of Physical Activity During School Recess: Impact of Playground Markings. *Frontiers in Public Health* 2018;**6**.<https://doi.org/10.3389/fpubh.2018.00283>

27. Baquet G, Ridgers ND, Blaes A, Aucouturier J, Van Praagh E, Berthoin S. Objectively assessed recess physical activity in girls and boys from high and low socioeconomic backgrounds. *BMC Public Health* 2014;**14**(1).<https://doi.org/10.1186/1471-2458-14-192>

28. Barbosa SC, Coledam DHC, Stabelini Neto A, Elias RGM, Oliveira ARD. School environment, sedentary behavior and physical activity in preschool children. *Revista Paulista de Pediatria* 2016;**34**(3):301-8.<https://doi.org/10.1016/j.rpped.2016.01.001>

29. Barton J, Sandercock G, Pretty J, Wood C. The effect of playground-and nature-based playtime interventions on physical activity and self-esteem in UK school children. *Int J Environ Health Res* 2015;**25**(2):196-206.<https://doi.org/10.1080/09603123.2014.915020>

30. Bates CR, Bohnert AM, Gerstein DE. Green schoolyards in low-income urban neighborhoods: Natural spaces for positive youth development outcomes. *Front Psychol* 2018;**9**(MAY).<https://doi.org/10.3389/fpsyg.2018.00805>

31. Benthroldo RS, Paravidino VB, Cunha DB, Mediano MFF, Sichieri R, Marques ES. Environment modification at school to promote physical activity among adolescents: a cluster randomized controlled trial. *Revista Brasileira de Epidemiologia* 2022;**25**.<https://doi.org/10.1590/1980-549720220019>

32. Berg S. Children’s Activity Levels in Different Playground Environments: An Observational Study in Four Canadian Preschools. *Early Childhood Education Journal* 2015;**43**(4):281-7.<https://doi.org/10.1007/s10643-014-0654-5>

33. Besenyi GM, Kaczynski AT, Stanis SAW, Vaughan KB. Demographic variations in observed energy expenditure across park activity areas. *Prev Med* 2013;**56**(1):79-81.<https://doi.org/10.1016/j.ypmed.2012.10.011>

34. Bjorgen K. Children's well-being and involvement in physically active outdoors play in a Norwegian kindergarten: Playful sharing of physical experiences. *Child Care in Practice* 2015;**21**(4):305-23.<https://doi.org/10.1080/13575279.2015.1051512>

35. Black IE, Menzel NN, Bungum TJ. The relationship among playground areas and physical activity levels in children. *J Pediatr Health Care* 2015;**29**(2):156-68.<https://doi.org/10.1016/j.pedhc.2014.10.001>

36. Blaes A, Ridgers ND, Aucouturier J, Van Praagh E, Berthoin S, Baquet G. Effects of a playwound marking intervention on school recess physical activity in French children. *Prev Med* 2013;**57**(5):580-4.<https://doi.org/10.1016/j.ypmed.2013.07.019>

37. Blatchford P, Baines E, Pellegrini A. The social context of school playground games: Sex and ethnic differences, and changes over time after entry to junior school. *Br J Dev Psychol* 2003;**21**(4):481-505.<https://doi.org/10.1348/026151003322535183>

38. Bohn-Goldbaum EE, Phongsavan P, Merom D, Rogers K, Kamalesh V, Bauman AE. Does playground improvement increase physical activity among children? A quasi-experimental study of a natural experiment. *J Environ Public Health* 2013;**2013**.<https://doi.org/10.1155/2013/109841>

39. Bohnert AM, Nicholson LM, Mertz L, Bates CR, Gerstein DE. Green schoolyard renovations in low-income urban neighborhoods: Benefits to students, schools, and the surrounding community. *Am J Community Psychol* 2022.<https://doi.org/10.1002/ajcp.12559>

40. Boldemann C, Dal H, Martensson F, et al. Preschool outdoor play environment may combine promotion of children's physical activity and sun protection. Further evidence from Southern Sweden and North Carolina. *Sci Sports* 2011;**26**(2):72-82.<https://doi.org/10.1016/j.scispo.2011.01.007>

41. Boonzajer Flaes SAM, Chinapaw MJM, Koolhaas CM, van Mechelen W, Verhagen EALM. More children more active: Tailored playgrounds positively affect physical activity levels amongst youth. *J Sci Med Sport* 2016;**19**(3):250-4.<https://doi.org/10.1016/j.jsams.2015.03.001>

42. Bourke TM, Sargisson RJ. A behavioral investigation of preference in a newly designed New Zealand playground. *American Journal of Play* 2014;**6**(3):370-91.<https://doi.org/10.18666/jpra-2023-10575>

43. Bower JK, Hales DP, Tate DF, Rubin DA, Benjamin SE, Ward DS. The childcare environment and children's physical activity. *Am J Prev Med* 2008;**34**(1):23-9.<https://doi.org/10.1016/j.amepre.2007.09.022>

44. Boyle DE, Marshall NL, Robeson WW. Gender at play: Fourth-grade girls and boys on the playground. *Am Behav Sci* 2003;**46**(10):1326-45.<https://doi.org/10.1177/0002764203046010004>

45. Brink LA, Nigg CR, Lampe SMR, Kingston BA, Mootz AL, van Vliet W. Influence of schoolyard renovations on children's physical activity: The Learning Landscapes Program. *Am J Public Health* 2010;**100**(9):1672-8.<https://doi.org/10.2105/AJPH.2009.178939>

46. Brown WH, Pfeiffer KA, McIver KL, Dowda M, Addy CL, Pate RR. Social and environmental factors associated with preschoolers' nonsedentary physical activity. *Child Dev* 2009;**80**(1):45-58.<https://doi.org/10.1111/j.1467-8624.2008.01245.x>

47. Brussoni M, Ishikawa T, Brunelle S, Herrington S. Landscapes for play: Effects of an intervention to promote nature-based risky play in early childhood centres. *J Environ Psychol* 2017;**54**:139-50.<https://doi.org/10.1016/j.jenvp.2017.11.001>

48. Buck C, Tkaczick T, Pitsiladis Y, et al. Objective Measures of the Built Environment and Physical Activity in Children: From Walkability to Moveability. *J Urban Health* 2015;**92**(1):24-38.<https://doi.org/10.1007/s11524-014-9915-2>

49. Burgi R, Tomatis L, Murer K, de Bruin ED. Localization of Physical Activity in Primary School Children Using Accelerometry and Global Positioning System. *PLoS One* 2015;**10**(11).<https://doi.org/10.1371/journal.pone.0142223>

50. Cardon G, Labarque V, Smits D, Bourdeaudhuij ID. Promoting physical activity at the pre-school playground: The effects of providing markings and play equipment. *Prev Med* 2009;**48**(4):335-40.<https://doi.org/10.1016/j.ypmed.2009.02.013>

51. Cardon G, Van Cauwenberghe E, Labarque V, Haerens L, De Bourdeaudhuij I. The contribution of preschool playground factors in explaining children's physical activity during recess. *Int J Behav Nutr Phys Act* 2008;**5**.<https://doi.org/10.1186/1479-5868-5-11>

52. Cerin E, Baranowski T, Barnett A, et al. Places where preschoolers are (in)active: An observational study on Latino preschoolers and their parents using objective measures. *Int J Behav Nutr Phys Act* 2016;**13**.<https://doi.org/10.1186/s12966-016-0355-0>

53. Chen B, Waters CN, Compier T, et al. Understanding physical activity and sedentary behaviour among preschool-aged children in Singapore: A mixed-methods approach. *BMJ Open* 2020;**10**(4).<https://doi.org/10.1136/bmjopen-2019-030606>

54. Christiansen LB, Toftager M, Boyle E, Kristensen PL, Troelsen J. Effect of a school environment intervention on adolescent adiposity and physical fitness. *Scand J Med Sci Sports* 2013;**23**(6):e381-e9.<https://doi.org/10.1111/sms.12088>

55. Christiansen LB, Toftager M, Pawlowski CS, Andersen HB, Ersboll AK, Troelsen J. Schoolyard upgrade in a randomized controlled study design-How are school interventions associated with adolescents' perception of opportunities and recess physical activity. *Health Educ Res* 2017;**32**(1):58-68.<https://doi.org/10.1093/her/cyw058>

56. Clarke KM. Benching playground loneliness: Exploring the meanings of the playground buddy bench. *International Electronic Journal of Elementary Education* 2018;**11**(1):9-21.<https://doi.org/10.26822/IEJEE.2018143930>

57. Clevenger KA, Erickson KT, Grady SC, Pfeiffer KA. Characterizing preschooler's outdoor physical activity: The comparability of schoolyard location- and activity type-based approaches. *Early Childhood Research Quarterly* 2021;**56**:139-48.<https://doi.org/10.1016/j.ecresq.2021.03.012>

58. Clevenger KA, Grady SC, Erickson K, Pfeiffer KA. Use of a spatiotemporal approach for understanding preschoolers’ playground activity. *Spatial and Spatio-temporal Epidemiology* 2020;**35**.<https://doi.org/10.1016/j.sste.2020.100376>

59. Cohen DA, Han B, Williamson S, et al. Playground features and physical activity in U.S. neighborhood parks. *Prev Med* 2020;**131**.<https://doi.org/10.1016/j.ypmed.2019.105945>

60. Colabianchi N, Kinsella AE, Coulton CJ, Moore SM. Utilization and physical activity levels at renovated and unrenovated school playgrounds. *Prev Med* 2009;**48**(2):140-3.<https://doi.org/10.1016/j.ypmed.2008.11.005>

61. Colabianchi N, Maslow AL, Swayampakala K. Features and amenities of school playgrounds: A direct observation study of utilization and physical activity levels outside of school time. *Int J Behav Nutr Phys Act* 2011;**8**.<https://doi.org/10.1186/1479-5868-8-32>

62. Connelly J-A, Manningham S, Champagne M. Factors related to energetic play during outdoor time in childcare centres. *Early Childhood Education Journal* 2021;**49**(3):441-9.<https://doi.org/10.1007/s10643-020-01088-8>

63. Copeland KA, Kendeigh CA, Saelens BE, Kalkwarf HJ, Sherman SN. Physical activity in child-care centers: Do teachers hold the key to the playground? *Health Educ Res* 2012;**27**(1):81-100.<https://doi.org/10.1093/her/cyr038>

64. Copeland KA, Khoury JC, Kalkwarf HJ. Child care center characteristics associated with preschoolers' physical activity. *Am J Prev Med* 2016;**50**(4):470-9.<https://doi.org/10.1016/j.amepre.2015.08.028>

65. Coplan RJ, Ooi LL, Rose-Krasnor L. Naturalistic observations of schoolyard social participation: Marker variables for socio-emotional functioning in early adolescence. *The Journal of Early Adolescence* 2015;**35**(5-6):628-50.<https://doi.org/10.1177/0272431614523134>

66. Cosco NG, Moore RC, Islam MZ. Behavior mapping: A method for linking preschool physical activity and outdoor design. *Med Sci Sports Exerc* 2010;**42**(3):513-9.<https://doi.org/10.1249/MSS.0b013e3181cea27a>

67. Crust L, McKenna J, Spence J, Thomas C, Evans D, Bishop D. The effects of playground markings on the physical self-perceptions of 10-11-year-old school children. *Physical Education and Sport Pedagogy* 2014;**19**(2):179-90.<https://doi.org/10.1080/17408989.2012.732565>

68. Czalczynska-Podolska M. The impact of playground spatial features on children's play and activity forms: An evaluation of contemporary playgrounds' play and social value. *J Environ Psychol* 2014;**38**:132-42.<https://doi.org/10.1016/j.jenvp.2014.01.006>

69. Da Silva BMS, Rieffe C, Frijns JHM, Sousa H, Monteiro L, Veiga G. Being Deaf in Mainstream Schools: The Effect of a Hearing Loss in Children’s Playground Behaviors. *Children* 2022;**9**(7).<https://doi.org/10.3390/children9071091>

70. Dalene KE, Anderssen SA, Ekelund U, Thorén AKH, Hansen BH, Kolle E. Permanent play facility provision is associated with children's time spent sedentary and in light physical activity during school hours: A cross-sectional study. *Preventive Medicine Reports* 2016;**4**:429-34.<https://doi.org/10.1016/j.pmedr.2016.08.011>

71. Delidou E, Matsouka O, Nikolaidis C. Influence of school playground size and equipment on the physical activity of students during recess. *European Physical Education Review* 2016;**22**(2):215-24.<https://doi.org/10.1177/1356336x15598790>

72. Dessing D, Pierik FH, Sterkenburg RP, van Dommelen P, Maas J, de Vries SI. Schoolyard physical activity of 6-11 year old children assessed by GPS and accelerometry. *International Journal of Behavioral Nutrition and Physical Activity* 2013;**10**.<https://doi.org/10.1186/1479-5868-10-97>

73. Dinkel D, Dev D, Guo Y, et al. Comparison of Urban and Rural Physical Activity and Outdoor Play Environments of Childcare Centers and Family Childcare Homes. *Fam Community Health* 2020;**43**(4):264-75.<https://doi.org/10.1097/FCH.0000000000000267>

74. Dinkel D, Snyder K, Patterson T, Warehime S, Kuhn M, Wisneski D. An exploration of infant and toddler unstructured outdoor play. *European Early Childhood Education Research Journal* 2019;**27**(2):257-71.<https://doi.org/10.1080/1350293X.2019.1579550>

75. Dudley DA, Cotton WG, Peralta LR, Winslade M. Playground activities and gender variation in objectively measured physical activity intensity in Australian primary school children: A repeated measures study. *BMC Public Health* 2018;**18**(1).<https://doi.org/10.1186/s12889-018-6005-5>

76. Dyment JE, Bell AC. Grounds for movement: Green school grounds as sites for promoting physical activity. *Health Educ Res* 2008;**23**(6):952-62.<https://doi.org/10.1093/her/cym059>

77. Dyment JE, Bell AC, Lucas AJ. The relationship between school ground design and intensity of physical activity. *Children's Geographies* 2009;**7**(3):261-76.<https://doi.org/10.1080/14733280903024423>

78. El-Kholy SA, Moustafa YM, Abou El-Ela MAS. Urban park design and children’s physical activity levels: an investigation of design characteristics of green areas and playgrounds. *Journal of Engineering and Applied Science* 2022;**69**(1).<https://doi.org/10.1186/s44147-022-00152-x>

79. Escalante Y, Backx K, Saavedra JM, García-Hermoso A, Domínguez AM. Relationship between daily physical activity, recess physical activity, age and sex in scholar of primary school, Spain. *Rev Esp Salud Publica* 2011;**85**(5):481-9.<https://doi.org/10.1590/s1135-57272011000500007>

80. Escalante Y, Backx K, Saavedra JM, Garcia-Hermoso A, Dominguez AM. Play area and physical activity in recess in primary schools. *Kinesiology* 2012;**44**(2):123-9

81. Escaron AL, Vega-Herrera C, Steers N, et al. Factors Associated With Leisure Time Physical Activity Among Schoolchildren in a Predominantly Latino Community. *J Sch Health* 2019;**89**(6):444-51.<https://doi.org/10.1111/josh.12755>

82. Eskola S, Tossavainen K, Bessems K, Sormunen M. Children’s perceptions of factors related to physical activity in schools. *Educational Research* 2018;**60**(4):410-26.<https://doi.org/10.1080/00131881.2018.1530948>

83. Eyre ELJ, Duncan MJ, Birch SL, Cox V. Environmental and school influences on physical activity in South Asian children from low socio-economic backgrounds: A qualitative study. *Journal of Child Health Care* 2015;**19**(3):345-58.<https://doi.org/10.1177/1367493513508845>

84. Fahlén J. Urgent expectations and silenced knowledge On spontaneous sport space as public health promoter and sport stimulator. *European Journal for Sport and Society* 2011;**8**(3):167-91.<https://doi.org/10.1080/16138171.2011.11687877>

85. Famelia R, Tsuda E, Bakhtiar S, Goodway JD. Relationships among perceived and actual motor skill competence and physical activity in Indonesian preschoolers. *Journal of Motor Learning and Development* 2018;**6**:S403-S23.<https://doi.org/10.1123/jmld.2016-0072>

86. Farley TA, Meriwether RA, Baker ET, Rice JC, Webber LS. Where do the children play? The influence of playground equipment on physical activity of children in free play. *Journal of Physical Activity and Health* 2008;**5**(2):319-31.<https://doi.org/10.1123/jpah.5.2.319>

87. Farley TA, Meriwether RA, Baker ET, Watkins LT, Johnson CC, Webber LS. Safe play spaces to promote physical activity in inner-city children: Results from a pilot study of an environmental intervention. *Am J Public Health* 2007;**97**(9):1625-31.<https://doi.org/10.2105/AJPH.2006.092692>

88. Fjortoft I. The natural environment as a playground for children: The impact of outdoor play activities in pre-primary school children. *Early Childhood Education Journal* 2001;**29**(2):111-7.<https://doi.org/10.1023/A:1012576913074>

89. Fjortoft I, Kristoffersen B, Sageie J. Children in schoolyards: Tracking movement patterns and physical activity in schoolyards using global positioning system and heart rate monitoring. *Landscape and Urban Planning* 2009;**93**(3-4):210-7.<https://doi.org/10.1016/j.landurbplan.2009.07.008>

90. Fjortoft I, Lofman O, Thoren KH. Schoolyard physical activity in 14-year-old adolescents assessed by mobile GPS and heart rate monitoring analysed by GIS. *Scandinavian Journal of Public Health* 2010;**38**(Suppl 5):28-37.<https://doi.org/10.1177/1403494810384909>

91. Flouri E, Midouhas E, Joshi H. The role of urban neighbourhood green space in children's emotional and behavioural resilience. *J Environ Psychol* 2014;**40**:179-86.<https://doi.org/10.1016/j.jenvp.2014.06.007>

92. Foweather L, Crotti M, Foulkes JD, et al. Foundational Movement Skills and Play Behaviors during Recess among Preschool Children: A Compositional Analysis. *Children-Basel* 2021;**8**(7).<https://doi.org/10.3390/children8070543>

93. Frost MC, Kuo ES, Harner LT, Landau KR, Baldassar K. Increase in physical activity sustained 1 year after playground intervention. *Am J Prev Med* 2018;**54**(5, Suppl 2):S124-S9.<https://doi.org/10.1016/j.amepre.2018.01.006>

94. Gil-Madrona P, Martínez-López M, Prieto-Ayuso A, et al. Contribution of public playgrounds to motor, social, and creative development and obesity reduction in children. *Sustainability (Switzerland)* 2019;**11**(14).<https://doi.org/10.3390/su11143787>

95. Gilmore S, Frederick LK, Santillan L, Locke J. The games they play: Observations of children with autism spectrum disorder on the school playground. *Autism* 2019;**23**(6):1343-53.<https://doi.org/10.1177/1362361318811987>

96. Graham M, Dixon K, Azevedo LB, Wright MD, Innerd A. A socio-ecological examination of the primary school playground: Primary school pupil and staff perceived barriers and facilitators to a physically active playground during break and lunch-times. *PLoS One* 2022;**17**(2 February).<https://doi.org/10.1371/journal.pone.0261812>

97. Graham M, Wright M, Azevedo LB, Macpherson T, Jones D, Innerd A. The school playground environment as a driver of primary school children's physical activity behaviour: A direct observation case study. *J Sports Sci* 2021;**39**(20):2266-78.<https://doi.org/10.1080/02640414.2021.1928423>

98. Grunseit AC, O'Hara BJ, Drayton B, et al. Ecological study of playground space and physical activity among primary school children. *BMJ open* 2020;**10**(6):e034586.<https://doi.org/10.1136/bmjopen-2019-034586>

99. Gu L. A post-occupancy playground study: relation between user behaviour and design parameters. *Journal of Urban Design* 2021;**26**(6):746-63.<https://doi.org/10.1080/13574809.2021.1930525>

100. Gubbels JS, Kremers SPJ, van Kann DHH, et al. Interaction Between Physical Environment, Social Environment, and Child Characteristics in Determining Physical Activity at Child Care. *Health Psychol* 2011;**30**(1):84-90.<https://doi.org/10.1037/a0021586>

101. Gustat J, Anderson CE, Slater SJ. Association of Playground “Playability” With Physical Activity and Energy Expenditure. *Prev Chronic Dis* 2023;**20**.<https://doi.org/10.5888/pcd20.220247>

102. Hamer M, Aggio D, Knock G, Kipps C, Shankar A, Smith L. Effect of major school playground reconstruction on physical activity and sedentary behaviour: Camden active spaces. *BMC Public Health* 2017;**17**(1).<https://doi.org/10.1186/s12889-017-4483-5>

103. Hammarsten M. What are Schoolchildren doing Out There? Children’s Perspectives on Aff ordances in Unedited Places. *Built Environ* 2021;**47**(2):186-205.<https://doi.org/10.2148/BENV.47.2.186>

104. Hannus A, Lees M, Magi K, et al. Perspectives of children and adolescents on the perceived determinants of physical activity during recess. *Psychol Health Med* 2018;**23**(8):1016-24.<https://doi.org/10.1080/13548506.2017.1417611>

105. Harrison F, van Sluijs EMF, Corder K, Jones A. School grounds and physical activity: Associations at secondary schools, and over the transition from primary to secondary schools. *Health and Place* 2016;**39**:34-42.<https://doi.org/10.1016/j.healthplace.2016.02.004>

106. Harten N, Olds T, Dollman J. The effects of gender, motor skills and play area on the free play activities of 8-11 year old school children. *Health and Place* 2008;**14**(3):386-93.<https://doi.org/10.1016/j.healthplace.2007.08.005>

107. Haug E, Torsheim T, Sallis JF, Samdal O. The characteristics of the outdoor school environment associated with physical activity. *Health Educ Res* 2010;**25**(2):248-56.<https://doi.org/10.1093/her/cyn050>

108. Haug E, Torsheim T, Samdal O. Physical environmental characteristics and individual interests as correlates of physical activity in Norwegian secondary schools: The health behaviour in school-aged children study. *Int J Behav Nutr Phys Act* 2008;**5**.<https://doi.org/10.1186/1479-5868-5-47>

109. Hazlehurst MF, Wolf KL, Simmons C, et al. Physical activity and social interaction assessments in schoolyard settings using the System for Observing Outdoor Play Environments in Neighborhood Schools (SOOPEN). *International Journal of Behavioral Nutrition and Physical Activity* 2023;**20**(1).<https://doi.org/10.1186/s12966-023-01483-5>

110. Hogman J, Augustsson C, Hedstrom P. Let's do those 60 minutes! Children's perceived landscape for daily physical activity. *Sport, Education and Society* 2020;**25**(4):395-408.<https://doi.org/10.1080/13573322.2019.1610374>

111. Howe CA, Clevenger KA, Plow B, Porter S, Sinha G. Using Video Direct Observation to Assess Children's Physical Activity During Recess. *Pediatr Exerc Sci* 2018;**30**(4):516-23.<https://doi.org/10.1123/pes.2017-0203>

112. Huang W, Luo J, Chen Y. Effects of Kindergarten, Family Environment, and Physical Activity on Children's Physical Fitness. *Frontiers in Public Health* 2022;**10**.<https://doi.org/10.3389/fpubh.2022.904903>

113. Huberty JL, Siahpush M, Beighle A, Fuhrmeister E, Silva P, Welk G. Ready for Recess: A Pilot Study to Increase Physical Activity in Elementary School Children. *J Sch Health* 2011;**81**(5):251-7.<https://doi.org/10.1111/j.1746-1561.2011.00591.x>

114. Hyndman B, Mahony L. Developing creativity through outdoor physical activities: A qualitative exploration of contrasting school equipment provisions. *Journal of Adventure Education and Outdoor Learning* 2018;**18**(3):242-56.<https://doi.org/10.1080/14729679.2018.1436078>

115. Hyndman B, Telford A. Should educators be 'wrapping school playgrounds in cotton wool' to encourage physical activity? Exploring primary and secondary students' voices from the school playground. *Australian Journal of Teacher Education* 2015;**40**(6):60-84.<https://doi.org/10.14221/ajte.2015v40n6.4>

116. Hyndman B, Telford A, Finch FC, Benson CA. Moving physical activity beyond the school classroom: A social-ecological insight for teachers of the facilitators and barriers to students' noncurricular physical activity. *Australian Journal of Teacher Education* 2012;**37**(2):1-25.<https://doi.org/10.14221/ajte.2012v37n2.2>

117. Ip P, Ho FK-W, Louie LH-T, et al. Childhood obesity and physical activity-friendly school environments. *The Journal of Pediatrics* 2017;**191**:110-6.<https://doi.org/10.1016/j.jpeds.2017.08.017>

118. James ME, Jianopoulos E, Ross T, Buliung R, Arbour-Nicitopoulos KP. Children’s Usage of Inclusive Playgrounds: A Naturalistic Observation Study of Play. *Int J Environ Res Public Health* 2022;**19**(20).<https://doi.org/10.3390/ijerph192013648>

119. Kamal AM, Gabr HS. Enhancing children's social and cognitive development through play space design. *International Journal of Architectural Research: Archnet-IJAR* 2023.<https://doi.org/10.1108/ARCH-05-2023-0119>

120. Kasari C, Locke J, Gulsrud A, Rotheram-Fuller E. Social networks and friendships at school: Comparing children with and without ASD. *J Autism Dev Disord* 2011;**41**(5):533-44.<https://doi.org/10.1007/s10803-010-1076-x>

121. Kimbro RT, Brooks-Gunn J, McLanahan S. Young children in urban areas: Links among neighborhood characteristics, weight status, outdoor play, and television watching. *Soc Sci Med* 2011;**72**(5):668-76.<https://doi.org/10.1016/j.socscimed.2010.12.015>

122. Kirkeby IM, Grangaard S. Can we build inclusion? 2016. p. 246-55.

123. Kjonniksen L, Wiium N, Fjortoft I. Affordances of School Ground Environments for Physical Activity: A Case Study on 10-and 12-Year-Old Children in a Norwegian Primary School. *Frontiers in Public Health* 2022;**10**.<https://doi.org/10.3389/fpubh.2022.773323>

124. Knowles ZR, Parnell D, Stratton G, Ridgers ND. Learning from the experts: Exploring playground experience and activities using a write and draw technique. *Journal of Physical Activity & Health* 2013;**10**(3):406-15.<https://doi.org/10.1123/jpah.10.3.406>

125. Lahuerta-Contell S, Molina-García J, Queralt A, Martínez-Bello VE. The role of preschool hours in achieving physical activity recommendations for preschoolers. *Children* 2021;**8**(2).<https://doi.org/10.3390/children8020082>

126. Larsen MS, Agerskov H. The importance of an outdoor playground for children with epilepsy and their family during and after hospitalization: A qualitative study of parents' experiences. *J Pediatr Nurs* 2022;**66**:e16-e21.<https://doi.org/10.1016/j.pedn.2022.07.001>

127. Lemberg GM, Riso EM, Fjortoft I, Kjonniksen L, Kull M, Mäestu E. School Children's Physical Activity and Preferred Activities during Outdoor Recess in Estonia: Using Accelerometers, Recess Observation, and Schoolyard Mapping. *Children-Basel* 2023;**10**(4).<https://doi.org/10.3390/children10040702>

128. Li J, Hestenes LL, Wang YC. Links between preschool children's social skills and observed pretend play in outdoor childcare environments. *Early Childhood Education Journal* 2016;**44**(1):61-8.<https://doi.org/10.1007/s10643-014-0673-2>

129. Loebach J, Cox A. Playing in '<i>The Backyard</i>': Environmental Features and Conditions of a Natural Playspace Which Support Diverse Outdoor Play Activities among Younger Children. *Int J Environ Res Public Health* 2022;**19**(19).<https://doi.org/10.3390/ijerph191912661>

130. Loftesnes JM. In the nature playground – initiation and evaluation of the project at 9 kindergartens. *Journal of Physical Education and Sport* 2021;**21**:542-8.<https://doi.org/10.7752/jpes.2021.s1060>

131. López-Fernández I, Molina-Jodar M, Garrido-González FJ, Pascual-Martos CA, Chinchilla JL, Carnero EA. Promoting physical activity at the school playground: a quasi-experimental intervention study. *Journal of Human Sport & Exercise* 2016;**11**(2):319-28.<https://doi.org/10.14198/jhse.2016.112.05>

132. Luchs A, Fikus M. Differently designed playgrounds and preschooler's physical activity play. *Early Child Development and Care* 2018;**188**(3):281-95.<https://doi.org/10.1080/03004430.2016.1213726>

133. Luke JJ, Brenkert S, Rivera N. Preschoolers’ social emotional learning in children’s museums and community playgrounds. *Journal of Early Childhood Research* 2022;**20**(2):229-41.<https://doi.org/10.1177/1476718X211059913>

134. Lund S, Riiser K, Londal K. Children's Experiences with Outdoor, Physically Active Play in After-School Programs ? *American Journal of Play* 2023;**15**(1):35-59

135. Lundy A, Trawick-Smith J. Effects of active outdoor play on preschool children's on-task classroom behavior. *Early Childhood Education Journal* 2021;**49**(3):463-71.<https://doi.org/10.1007/s10643-020-01086-w>

136. Määttä S, Gubbels J, Ray C, et al. Children's physical activity and the preschool physical environment: The moderating role of gender. *Early Childhood Research Quarterly* 2019;**47**:39-48.<https://doi.org/10.1016/j.ecresq.2018.10.008>

137. MacArthur B, Coe D, Sweet A, Raynor H. Active videogaming compared to unstructured, outdoor play in young children: Percent time in moderate- to vigorous-intensity physical activity and estimated energy expenditure. *Games for Health* 2014;**3**(6):388-94.<https://doi.org/10.1089/g4h.2014.0017>

138. Mahony L, Hyndman B, Nutton G, Smith S, Te Ava A. Monkey bars, noodles and hay bales: a comparative analysis of social interaction in two school ground contexts. *International Journal of Play* 2017;**6**(2):166-76.<https://doi.org/10.1080/21594937.2017.1348319>

139. Marquet O, Hipp JA, Alberico C, et al. Park use preferences and physical activity among ethnic minority children in low-income neighborhoods in New York City. *Urban Forestry & Urban Greening* 2019;**38**:346-53.<https://doi.org/10.1016/j.ufug.2019.01.018>

140. Martinez-Andres M, Bartolome-Gutierrez R, Rodriguez-Martin B, Pardo-Guijarro MJ, Martinez-Vizcaino V. "Football is a boys' game": Children's perceptions about barriers for physical activity during recess time. *International Journal of Qualitative Studies on Health and Well-being* 2017;**12**(sup2).<https://doi.org/10.1080/17482631.2017.1379338>

141. Massey WV, Perez D, Neilson L, Thalken J, Szarabajko A. Observations from the playground: Common problems and potential solutions for school-based recess. *Health Educ J* 2021;**80**(3):313-26.<https://doi.org/10.1177/0017896920973691>

142. McLaughlin E, O'Donoghue PG. Activity profile of primary school children in the playground. *Journal of Human Movement Studies* 2002;**42**(2):91-108

143. McWhannell N, Triggs C, Moss S. Perceptions and measurement of playtime physical activity in English primary school children: The influence of socioeconomic status. *European Physical Education Review* 2019;**25**(2):438-55.<https://doi.org/10.1177/1356336X17743048>

144. Mertens L, Van Cauwenberg J, Veitch J, Deforche B, Van Dyck D. Differences in park characteristic preferences for visitation and physical activity among adolescents: A latent class analysis. *PLoS One* 2019;**14**(3).<https://doi.org/10.1371/journal.pone.0212920>

145. Miller LJ, Schoen SA, Camarata SM, et al. Play in natural environments: A pilot study quantifying the behavior of children on playground equipment. *Journal of Occupational Therapy, Schools, and Early Intervention* 2017;**10**(3):213-31.<https://doi.org/10.1080/19411243.2017.1325818>

146. Moges T, Gebremichael B, Shiferaw S, Yirgu R. Is inadequate play area in schools associated with overweight among students in Addis Ababa, Ethiopia? A comparative cross-sectional study. *Epidemiology and health* 2018;**40**:e2018017.<https://doi.org/10.4178/epih.e2018017>

147. Molenberg FJM, Noordzij JM, Burdorf A, van Lenthe FJ. New physical activity spaces in deprived neighborhoods: Does it change outdoor play and sedentary behavior? A natural experiment. *Health & Place* 2019;**58**.<https://doi.org/10.1016/j.healthplace.2019.102151>

148. Moore D, Morrissey A-M, Robertson N. 'i feel like i'm getting sad there': Early childhood outdoor playspaces as places for children's wellbeing. *Early Child Development and Care* 2019:No-Specified.<https://doi.org/10.1080/03004430.2019.1651306>

149. Moreira M, Cordovil R, Lopes F, Da Silva BMS, Veiga G. The Relationship between the Quality of Kindergartens’ Outdoor Physical Environment and Preschoolers’ Social Functioning. *Education Sciences* 2022;**12**(10).<https://doi.org/10.3390/educsci12100661>

150. Mota J, Silva P, Santos MP, Ribeiro JC, Oliveira J, Duarte JA. Physical activity and school recess time: Differences between the sexes and the relationship between children's playground physical activity and habitual physical activity. *J Sports Sci* 2005;**23**(3):269-75.<https://doi.org/10.1080/02640410410001730124>

151. Nasar JL, Holloman CH. Playground Characteristics to Encourage Children to Visit and Play. *Journal of Physical Activity & Health* 2013;**10**(8):1201-8.<https://doi.org/10.1123/jpah.10.8.1201>

152. Ndhlovu S, Varea V. Primary school playgrounds as spaces of inclusion/exclusion in New South Wales, Australia. *Education 3-13* 2018;**46**(5):494-505.<https://doi.org/10.1080/03004279.2016.1273251>

153. Neshteruk CD, Mazzucca S, Ostbye T, Ward DS. The physical environment in family childcare homes and children's physical activity. *Child Care Health Dev* 2018;**44**(5):746-52.<https://doi.org/10.1111/cch.12578>

154. Ng M, Rosenberg M, Thornton A, et al. The effect of upgrades to childcare outdoor spaces on preschoolers’ physical activity: Findings from a natural experiment. *Int J Environ Res Public Health* 2020;**17**(2).<https://doi.org/10.3390/ijerph17020468>

155. Nicaise V, Kahan D, Reuben K, Sallis JF. Evaluation of a Redesigned Outdoor Space on Preschool Children's Physical Activity During Recess. *Pediatr Exerc Sci* 2012;**24**(4):507-18.<https://doi.org/10.1123/pes.24.4.507>

156. Nicaise V, Kahan D, Sallis JF. Correlates of moderate-to-vigorous physical activity among preschoolers during unstructured outdoor play periods. *Prev Med* 2011;**53**(4-5):309-15.<https://doi.org/10.1016/j.ypmed.2011.08.018>

157. Nichol ME, Pickett W, Janssen I. Associations Between School Recreational Environments and Physical Activity. *J Sch Health* 2009;**79**(6):247-54.<https://doi.org/10.1111/j.1746-1561.2009.00406.x>

158. Nielsen G, Bugge A, Hermansen B, Svensson J, Andersen LB. School playground facilities as a determinant of children's daily activity: A cross-sectional study of Danish primary school children. *Journal of Physical Activity & Health* 2012;**9**(1):104-14.<https://doi.org/10.1123/jpah.9.1.104>

159. Nielsen G, Taylor R, Williams S, Mann J. Permanent play facilities in school playgrounds as a determinant of children's activity. *Journal of Physical Activity and Health* 2010;**7**(4):490-6.<https://doi.org/10.1123/jpah.7.4.490>

160. Nobre JNP, Morais RLD, Prat BV, et al. Physical environmental opportunities for active play and physical activity level in preschoolers: a multicriteria analysis. *BMC Public Health* 2022;**22**(1).<https://doi.org/10.1186/s12889-022-12750-8>

161. Olesen LG, Kristensen PL, Korsholm L, Froberg K. Physical activity in children attending preschools. *Pediatrics* 2013;**132**(5):e1310-e8.<https://doi.org/10.1542/peds.2012-3961>

162. Oliveira P, Clemente FM, Martins FML. Assessment of interactions at children playgrounds using network measures: An exploratory study based on graph theory. *Journal of Physical Education and Sport* 2016;**16**:1012-6.<https://doi.org/10.7752/jpes.2016.s2160>

163. Oreskovic NM, Perrin JM, Robinson AI, et al. Adolescents' use of the built environment for physical activity. *BMC Public Health* 2015;**15**(1).<https://doi.org/10.1186/s12889-015-1596-6>

164. Ozdemir A, Yilmaz O. Assessment of outdoor school environments and physical activity in Ankara's primary schools. *J Environ Psychol* 2008;**28**(3):287-300.<https://doi.org/10.1016/j.jenvp.2008.02.004>

165. Pagels P, Raustorp A, De Leon AP, Martensson F, Kylin M, Boldemann C. A repeated measurement study investigating the impact of school outdoor environment upon physical activity across ages and seasons in Swedish second, fifth and eighth graders. *BMC Public Health* 2014;**14**.<https://doi.org/10.1186/1471-2458-14-803>

166. Pagels P, Wester U, Martensson F, et al. Pupils' use of school outdoor play settings across seasons and its relation to sun exposure and physical activity. *Photodermatology Photoimmunology & Photomedicine* 2020;**36**(5):365-72.<https://doi.org/10.1111/phpp.12558>

167. Palmer KK, Farquhar JM, Chinn KM, Robinson LE. Are Gross Motor Skill Interventions an Equitable Replacement for Outdoor Free Play Regarding Children's Physical Activity? *Am J Health Promot* 2022;**36**(4):643-50.<https://doi.org/10.1177/08901171211063261>

168. Parrish A-M, Iverson D, Russell K, Yeatman H. Observing Children's Playground Activity Levels at 13 Illawarra Primary Schools Using CAST2. *Journal of Physical Activity & Health* 2009;**6**:S89-S96.<https://doi.org/10.1123/jpah.6.s1.s89>

169. Parrish A-M, Yeatman H, Iverson D, Russell K. Using interviews and peer pairs to better understand how school environments affect young children's playground physical activity levels: A qualitative study. *Health Educ Res* 2012;**27**(2):269-80.<https://doi.org/10.1093/her/cyr049>

170. Pawlowski CS, Andersen HB, Arvidsen J, Schipperijn J. Changing recess geographies: children’s perceptions of a schoolyard renovation project promoting physical activity. *Children's Geographies* 2019;**17**(6):664-75.<https://doi.org/10.1080/14733285.2019.1582754>

171. Pawlowski CS, Andersen HB, Schipperijn J. Difference in outdoor time and physical activity during recess after schoolyard renewal for the least-active children. *Journal of Physical Activity & Health* 2020;**17**(10):968-76.<https://doi.org/10.1123/jpah.2019-0270>

172. Pawlowski CS, Andersen HB, Tjomhoj-Thomsen T, Troelsen J, Schipperjin J. Space, body, time and relationship experiences of recess physical activity: a qualitative case study among the least physical active schoolchildren. *BMC Public Health* 2016;**16**.<https://doi.org/10.1186/s12889-015-2687-0>

173. Pawlowski CS, Andersen HB, Troelsen J, Schipperijn J. Children's Physical Activity Behavior during School Recess: A Pilot Study Using GPS, Accelerometer, Participant Observation, and Go-Along Interview. *PLoS One* 2016;**11**(2).<https://doi.org/10.1371/journal.pone.0148786>

174. Pawlowski CS, Schipperijn J, Tjornhoj-Thomsen T, Troelsen J. Giving children a voice: Exploring qualitative perspectives on factors influencing recess physical activity. *European Physical Education Review* 2018;**24**(1):39-55.<https://doi.org/10.1177/1356336x16664748>

175. Pawlowski CS, Schmidt T, Nielsen JV, Troelsen J, Schipperijn J. Will the children use it?—A RE-AIM evaluation of a local public open space intervention involving children from a deprived neighbourhood. *Eval Program Plann* 2019;**77**.<https://doi.org/10.1016/j.evalprogplan.2019.101706>

176. Pawlowski CS, Tjornhoj-Thomsen T, Schipperijn J, Troelsen J. Barriers for recess physical activity: a gender specific qualitative focus group exploration. *BMC Public Health* 2014;**14**.<https://doi.org/10.1186/1471-2458-14-639>

177. Pawlowski CS, Veitch J, Andersen HB, Ridgers ND. Designing activating schoolyards: Seen from the girls’ viewpoint. *Int J Environ Res Public Health* 2019;**16**(19).<https://doi.org/10.3390/ijerph16193508>

178. Peden ME, Jones R, Costa S, Ellis Y, Okely AD. Relationship between children's physical activity, sedentary behavior, and childcare environments: A cross sectional study. *Preventive Medicine Reports* 2017;**6**:171-6.<https://doi.org/10.1016/j.pmedr.2017.02.017>

179. Pereira S, Reyes A, Moura-Dos-Santos MA, et al. Why are children different in their moderate-to-vigorous physical activity levels? A multilevel analysis. *J Pediatr (Rio J)* 2020;**96**(2):225-32.<https://doi.org/10.1016/j.jped.2018.10.013>

180. Poulos A, Wilson K, Lanza K, Vanos J. A direct observation tool to measure interactions between shade, nature, and children's physical activity: SOPLAY-SN. *Int J Behav Nutr Phys Act* 2022;**19**.<https://doi.org/10.1186/s12966-022-01355-4>

181. Powell E, Woodfield LA, Nevill AAM. Children’s physical activity levels during primary school break times: A quantitative and qualitative research design. *European Physical Education Review* 2016;**22**(1):82-98.<https://doi.org/10.1177/1356336X15591135>

182. Pratt B, Hartshorne NS, Mullens P, Schilling ML, Fuller S, Pisani E. Effect of Playground Environments on the Physical Activity of Children With Ambulatory Cerebral Palsy. *Pediatr Phys Ther* 2016;**28**(4):475-82.<https://doi.org/10.1097/pep.0000000000000318>

183. Prellwitz M, Skar L. Usability of playgrounds for children with different abilities. *Occup Ther Int* 2007;**14**(3):144-55.<https://doi.org/10.1002/oti.230>

184. Quigg R, Gray A, Reeder AI, Holt A, Waters DL. Using accelerometers and GPS units to identify the proportion of daily physical activity located in parks with playgrounds in New Zealand children. *Prev Med* 2010;**50**(5-6):235-40.<https://doi.org/10.1016/j.ypmed.2010.02.002>

185. Raney MA, Bowers AL, Rissberger AL. Recess Behaviors of Urban Children 16 Months After a Green Schoolyard Renovation. *Journal of Physical Activity & Health* 2021;**18**(5):563-70.<https://doi.org/10.1123/jpah.2020-0280>

186. Raney MA, Daniel E, Jack N. Impact of urban schoolyard play zone diversity and nature-based design features on unstructured recess play behaviors. *Landscape and Urban Planning* 2023;**230**.<https://doi.org/10.1016/j.landurbplan.2022.104632>

187. Raney MA, Hendry CF, Yee SA. Physical activity and social behaviors of urban children in green playgrounds. *Am J Prev Med* 2019;**56**(4):522-9.<https://doi.org/10.1016/j.amepre.2018.11.004>

188. Reimers AK, Knapp G. Playground usage and physical activity levels of children based on playground spatial features. *Journal of Public Health (Germany)* 2017;**25**(6):661-9.<https://doi.org/10.1007/s10389-017-0828-x>

189. Reimers AK, Schoeppe S, Demetriou Y, Knapp G. Physical activity and outdoor play of children in public playgrounds—do gender and social environment matter? *Int J Environ Res Public Health* 2018;**15**(7).<https://doi.org/10.3390/ijerph15071356>

190. Remmers T, Van Kann D, Thijs C, de Vries S, Kremers S. Playability of school-environments and after-school physical activity among 8-11 year-old children: Specificity of time and place. *Int J Behav Nutr Phys Act* 2016;**13**.<https://doi.org/10.1186/s12966-016-0407-5>

191. Ridgers ND, Stratton G. Physical Activity During School Recess: The Liverpool Sporting Playgrounds Project. *Pediatr Exerc Sci* 2005;**17**(3):281.<https://doi.org/10.1123/pes.17.3.281>

192. Ridgers ND, Stratton G, Fairclough SJ. Twelve-Month Effects of a Playground Intervention on Children’s Morning and Lunchtime Recess Physical Activity Levels. *Journal of Physical Activity and Health* 2010;**7**:167-75.<https://doi.org/10.1123/jpah.7.2.167>

193. Ridgers ND, Stratton G, Fairclough SJ, Twisk JWR. Long-term effects of a playground markings and physical structures on children's recess physical activity levels. *Prev Med* 2007;**44**(5):393-7.<https://doi.org/10.1016/j.ypmed.2007.01.009>

194. Ridgers ND, Stratton G, Fairclough SJ, Twisk JWR. Children's physical activity levels during school recess: A quasi-experimental intervention study. *Int J Behav Nutr Phys Act* 2007;**4**.<https://doi.org/10.1186/1479-5868-4-19>

195. Ristianti NS, Widjajanti R, editors. The effectiveness of inclusive playground usage for children through behavior-setting approach in Tembalang, Semarang city2020.

196. Rivera E, Timperio A, Loh VHY, Deforche B, Veitch J. Critical factors influencing adolescents’ active and social park use: A qualitative study using walk-along interviews. *Urban Forestry and Urban Greening* 2021;**58**.<https://doi.org/10.1016/j.ufug.2020.126948>

197. Rivera E, Timperio A, Loh VHY, Deforche B, Veitch J. Important park features for encouraging park visitation, physical activity and social interaction among adolescents: A conjoint analysis. *Health & Place* 2021;**70**.<https://doi.org/10.1016/j.healthplace.2021.102617>

198. Roemmich JN, Beeler JE, Johnson L. A microenvironment approach to reducing sedentary time and increasing physical activity of children and adults at a playground. *Preventive Medicine: An International Journal Devoted to Practice and Theory* 2014;**62**:108-12.<https://doi.org/10.1016/j.ypmed.2014.01.018>

199. Rydenstam T, Fell T, Buli BG, King AC, Balter K. Using citizen science to understand the prerequisites for physical activity among adolescents in low socioeconomic status neighborhoods - The NESLA study. *Health & Place* 2020;**65**.<https://doi.org/10.1016/j.healthplace.2020.102387>

200. Safari H, Khaftani PS. The Role of Green Playground on Social Interactions of Children (Case Study: Pasdaran Park in Bandar Anzali). *Tehnicki Glasnik* 2022;**16**(4):464-70.<https://doi.org/10.31803/tg-20220114174057>

201. Sando OJ. The outdoor environment and children’s health: a multilevel approach. *International Journal of Play* 2019;**8**(1):39-52.<https://doi.org/10.1080/21594937.2019.1580336>

202. Smith L, Gardner B, Aggio D, Hamer M. Association between participation in outdoor play and sport at 10years old with physical activity in adulthood. *Prev Med* 2015;**74**:31-5.<https://doi.org/10.1016/j.ypmed.2015.02.004>

203. Smith WR, Moore R, Cosco N, et al. Increasing Physical Activity in Childcare Outdoor Learning Environments: The Effect of Setting Adjacency Relative to Other Built Environment and Social Factors. *Environ Behav* 2016;**48**(4):550-78.<https://doi.org/10.1177/0013916514551048>

204. Soderstrom M, Boldemann C, Sahlin U, Martensson F, Raustorp A, Blennow M. The quality of the outdoor environment influences childrens health-A cross-sectional study of preschools. *Acta Paediatr* 2013;**102**(1):83-91.<https://doi.org/10.1111/apa.12047>

205. Stanton-Chapman TL, Schmidt EL. How Do the Children Play? The Influence of Playground Type on Children’s Play Styles. *Front Psychol* 2021;**12**.<https://doi.org/10.3389/fpsyg.2021.703940>

206. Stellino MB, Sinclair C. Examination of children's recess physical activity patterns using the activities for daily living-playground participation (ADL-PP) instrument. *Journal of Teaching in Physical Education* 2014;**33**(2):282-96.<https://doi.org/10.1123/jtpe.2013-0156>

207. Stephens RL, Xu Y, Lesesne CA, et al. Relationship between child care centers' compliance with physical activity regulations and children's physical activity, New York city, 2010. *Prev Chronic Dis* 2014;**11**(10).<https://doi.org/10.5888/pcd11.130432>

208. Stevens WR, Borchard JM, Sleeper P, et al. Inclusive community playgrounds benefit typically developing children: An objective analysis of physical activity. *Frontiers in Sports and Active Living* 2023;**4**.<https://doi.org/10.3389/fspor.2022.1100574>

209. Storli R, Hagen TL. Affordances in outdoor environments and children's physically active play in pre-school. *European Early Childhood Education Research Journal* 2010;**18**(4):445-56.<https://doi.org/10.1080/1350293X.2010.525923>

210. Storli R, Hansen Sandseter EB. Children's play, well-being and involvement: how children play indoors and outdoors in Norwegian early childhood education and care institutions. *International Journal of Play* 2019;**8**(1):65-78.<https://doi.org/10.1080/21594937.2019.1580338>

211. Stratton G. Promoting children's physical activity in primary school: An intervention study using playground markings. *Ergonomics* 2000;**43**(10):1538-46.<https://doi.org/10.1080/001401300750003961>

212. Stratton G, Leonard J. The effects of playground markings on the energy expenditure of 5-7-year-old school children. *Pediatr Exerc Sci* 2002;**14**(2):170-80.<https://doi.org/10.1123/pes.14.2.170>

213. Stratton G, Mullan E. The effect of multicolor playground markings on children's physical activity level during recess. *Preventive Medicine: An International Journal Devoted to Practice and Theory* 2005;**41**(5-6):828-33.<https://doi.org/10.1016/j.ypmed.2005.07.009>

214. Sumiya M, Nonaka T. Does the Spatial Layout of a Playground Affect the Play Activities in Young Children? A Pilot Study. *Front Psychol* 2021;**12**.<https://doi.org/10.3389/fpsyg.2021.627052>

215. Szeszulski J, Lorenzo E, Todd M, et al. Early Care and Education Center Environmental Factors Associated with Product- and Process-Based Locomotor Outcomes in Preschool-Age Children. *Int J Environ Res Public Health* 2022;**19**(4).<https://doi.org/10.3390/ijerph19042208>

216. Talarowski M, Cohen DA, Williamson S, Han B. Innovative playgrounds: Use, physical activity, and implications for health. *Public Health* 2019;**174**:102-9.<https://doi.org/10.1016/j.puhe.2019.06.002>

217. Tandon PS, Downing KL, Saelens BE, Christakis DA. Two approaches to increase physical activity for preschool children in child care centers: A matched-pair cluster-randomized trial. *Int J Environ Res Public Health* 2019;**16**(20).<https://doi.org/10.3390/ijerph16204020>

218. Tandon PS, Saelens BE, Christakis DA. Active play opportunities at child care. *Pediatrics* 2015;**135**(6):e1425-e31.<https://doi.org/10.1542/peds.2014-2750>

219. Tay GWN, Chan MJ, Kembhavi G, et al. Children's perceptions of factors influencing their physical activity: A focus group study on primary school children. *International Journal of Qualitative Studies on Health and Well-being* 2021;**16**(1).<https://doi.org/10.1080/17482631.2021.1980279>

220. Taylor RW, Farmer VL, Cameron SL, Meredith-Jones K, Williams SM, Mann JI. School playgrounds and physical activity policies as predictors of school and home time activity. *Int J Behav Nutr Phys Act* 2011;**8**.<https://doi.org/10.1186/1479-5868-8-38>

221. Taylor SL, Curry WB, Knowles ZR, Noonan RJ, McGrane B, Fairclough SJ. Predictors of segmented school day physical activity and sedentary time in children from a northwest England low-income community. *Int J Environ Res Public Health* 2017;**14**(5).<https://doi.org/10.3390/ijerph14050534>

222. Thornton CM, Cain KL, Conway TL, et al. Relation of Adolescents' Physical Activity to After-School Recreation Environment. *Journal of Physical Activity & Health* 2017;**14**(5):382-8.<https://doi.org/10.1123/jpah.2016-0365>

223. Tortella P, Haga M, Lorås H, Fumagalli GF, Sigmundsson H. Effects of Free Play and Partly Structured Playground Activity on Motor Competence in Preschool Children: A Pragmatic Comparison Trial. *Int J Environ Res Public Health* 2022;**19**(13).<https://doi.org/10.3390/ijerph19137652>

224. Tortella P, Haga M, Loras H, Sigmundsson H, Fumagalli G. Motor skill development in Italian pre-school children induced by structured activities in a specific playground. *PLoS One* 2016;**11**(7).<https://doi.org/10.1371/journal.pone.0160244>

225. True L, Pfeiffer KA, Dowda M, et al. Motor competence and characteristics within the preschool environment. *J Sci Med Sport* 2017;**20**(8):751-5.<https://doi.org/10.1016/j.jsams.2016.11.019>

226. Tucker P, Vanderloo LM, Burke SM, Irwin JD, Johnson AM. Prevalence and influences of preschoolers' sedentary behaviors in early learning centers: A cross-sectional study. *BMC Pediatr* 2015;**15**(1).<https://doi.org/10.1186/s12887-015-0441-5>

227. Van Cauwenberghe E, De Bourdeaudhuij I, Maes L, Cardon G. Efficacy and feasibility of lowering playground density to promote physical activity and to discourage sedentary time during recess at preschool: A pilot study. *Preventive Medicine: An International Journal Devoted to Practice and Theory* 2012;**55**(4):319-21.<https://doi.org/10.1016/j.ypmed.2012.07.014>

228. van Dijk-Wesselius JE, Maas J, Hovinga D, van Vugt M, van den Berg AE. The impact of greening schoolyards on the appreciation, and physical, cognitive and social-emotional well-being of schoolchildren: A prospective intervention study. *Landscape and Urban Planning* 2018;**180**:15-26.<https://doi.org/10.1016/j.landurbplan.2018.08.003>

229. Van Dyck D, Timmerman C, Hermida A, et al. Physical activity during recess in elementary schoolchildren in Belgium and Ecuador: The role of the physical environment at school. *J Sports Sci* 2022;**40**(13):1476-85.<https://doi.org/10.1080/02640414.2022.2086521>

230. Van Kann DHH, de Vries SI, Schipperijn J, de Vries NK, Jansen MWJ, Kremers SPJ. Schoolyard Characteristics, Physical Activity, and Sedentary Behavior: Combining GPS and Accelerometry. *J Sch Health* 2016;**86**(12):913-21.<https://doi.org/10.1111/josh.12459>

231. Van Kann DHH, de Vries SI, Schipperijn J, de Vries NK, Jansen MWJ, Kremers SPJ. A Multicomponent Schoolyard Intervention Targeting Children's Recess Physical Activity and Sedentary Behavior: Effects After 1 Year. *Journal of Physical Activity & Health* 2016;**14**(11):866-75.<https://doi.org/10.1123/jpah.2015-0702>

232. Vanderloo LM, Tucker P, Johnson AM, Holmes JD. Physical activity among preschoolers during indoor and outdoor childcare play periods. *Applied Physiology, Nutrition and Metabolism* 2013;**38**(11):1173-5.<https://doi.org/10.1139/apnm-2013-0137>

233. Veiga G, de Leng W, Cachucho R, et al. Social competence at the playground: Preschoolers during recess. *Infant and Child Development* 2017;**26**(1):No-Specified.<https://doi.org/10.1002/icd.1957>

234. Veiga G, Ketelaar L, De Leng W, et al. Alone at the playground. *Eur J Dev Psychol* 2017;**14**(1):44-61.<https://doi.org/10.1080/17405629.2016.1145111>

235. Veitch J, Salmon J, Crawford D, et al. The REVAMP natural experiment study: The impact of a play-scape installation on park visitation and park-based physical activity. *Int J Behav Nutr Phys Act* 2018;**15**.<https://doi.org/10.1186/s12966-017-0625-5>

236. Virji-Babul N, Hovorka R, Jobling A. Playground dynamics: Perceptual-motor behaviour and peer interactions of young children with Down syndrome. *Journal on Developmental Disabilities* 2006;**12**(1, Suppl 2):29-44

237. Webster EK, Kepper MM, Saha S, et al. Painted playgrounds for preschoolers’ physical activity and fundamental motor skill improvement: a randomized controlled pilot trial of effectiveness. *BMC Pediatr* 2023;**23**(1).<https://doi.org/10.1186/s12887-023-04260-2>

238. Willenberg LJ, Ashbolt R, Holland D, et al. Increasing school playground physical activity: A mixed methods study combining environmental measures and children's perspectives. *J Sci Med Sport* 2010;**13**(2):210-6.<https://doi.org/10.1016/j.jsams.2009.02.011>

239. Wishart L, Cabezas-Benalcázar C, Morrissey AM, Versace VL. Traditional vs naturalised design: a comparison of affordances and physical activity in two preschool playscapes. *Landscape Research* 2019;**44**(8):1031-49.<https://doi.org/10.1080/01426397.2018.1551524>

240. Wood C, Gladwell V, Barton J. A repeated measures experiment of school playing environment to increase physical activity and enhance self-esteem in UK school children. *PLoS One* 2014;**9**(9).<https://doi.org/10.1371/journal.pone.0108701>

241. Wood C, Hall K. Physical education or playtime: Which is more effective at promoting physical activity in primary school children? *BMC Res Notes* 2015;**8**(1).<https://doi.org/10.1186/s13104-015-0979-1>

242. Woods AM, Graber KC, Daum DN, Gentry C. Young school children's recess physical activity: Movement patterns and preferences. *Journal of Teaching in Physical Education* 2015;**34**(3):496-516.<https://doi.org/10.1123/jtpe.2014-0048>

243. Yang JT, Chen CI, Zheng MC. Elevating Children’s Play Experience: A Design Intervention to Enhance Children’s Social Interaction in Park Playgrounds. *Sustainability (Switzerland)* 2023;**15**(8).<https://doi.org/10.3390/su15086971>

244. Yildirim M, Arundell L, Cerin E, et al. What helps children to move more at school recess and lunchtime? Mid-intervention results from Transform-Us! cluster-randomised controlled trial. *Br J Sports Med* 2014;**48**(3):271-7.<https://doi.org/10.1136/bjsports-2013-092466>

245. Yuill N, Strieth S, Roake C, Aspden R, Todd B. Brief report: Designing a playground for children with autistic spectrum disorders - Effects on playful peer interactions. *J Autism Dev Disord* 2007;**37**(6):1192-6.<https://doi.org/10.1007/s10803-006-0241-8>

246. Zask A, van Beurden E, Barnett L, Brooks LO, Dietrich UC. Active school playgrounds-myth or reality? Results of the "Move It Groove It" project. *Preventive Medicine: An International Journal Devoted to Practice and Theory* 2001;**33**(5):402-8.<https://doi.org/10.1006/pmed.2001.0905>

247. Zumbrunn S, Doll B, Dooley K, LeClair C, Wimmer C. Assessing student perceptions of positive and negative social interactions in specific school settings. *International Journal of School & Educational Psychology* 2013;**1**(2):82-93.<https://doi.org/10.1080/21683603.2013.803001>
